# Regulation and clinical manifestations of gluconeogenesis dysfunction

**DOI:** 10.1038/s44324-026-00120-6

**Published:** 2026-07-21

**Authors:** Natalia Bobok

**Affiliations:** INSTYTUTUM AG, Zug, Switzerland

**Keywords:** Biochemistry, Cell biology, Endocrinology, Physiology

## Abstract

The liver is the principal organ responsible for maintaining systemic glucose homeostasis through the integrated regulation of gluconeogenesis, glycogenolysis, glycogenesis, and glycolysis. As fasting progresses and hepatic glycogen stores decline, gluconeogenesis becomes the dominant source of endogenous glucose production required to preserve euglycemia. This review summarizes the regulation of gluconeogenesis and discusses the clinical manifestations, diagnostic implications, and therapeutic relevance of gluconeogenic dysfunction across diverse disease states.

## Introduction

**Fig. 1:**
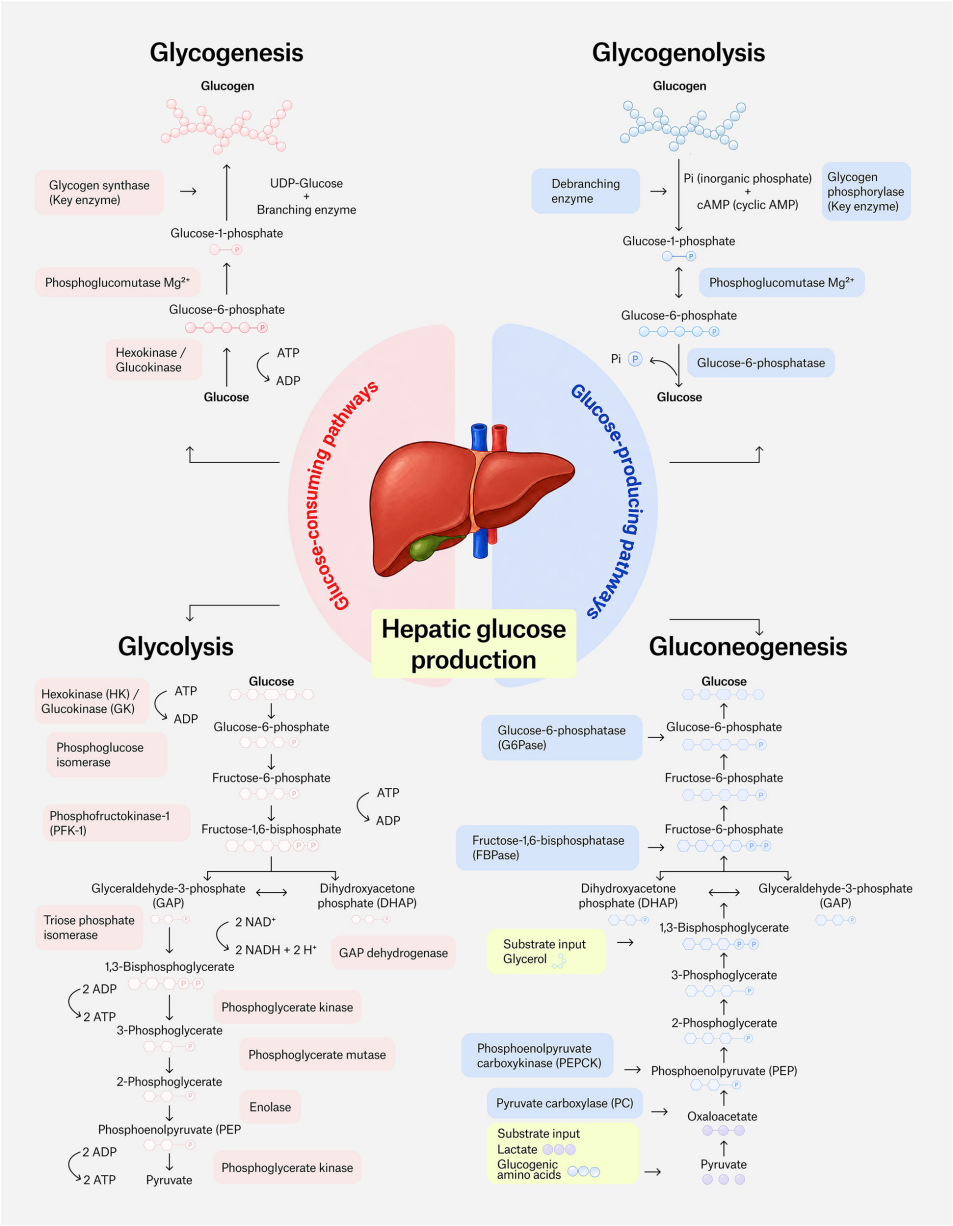
Integration of glycogenesis, glycogenolysis, glycolysis, and gluconeogenesis in hepatic glucose production. The figure illustrates the four major hepatic pathways that collectively regulate systemic glucose homeostasis. Glycogenesis stores glucose as glycogen, whereas glycogenolysis mobilizes glycogen reserves to generate glucose. Glycolysis utilizes glucose for energy production through its conversion to pyruvate. In contrast, gluconeogenesis synthesizes glucose from non-carbohydrate precursors, including glycerol, lactate, and glucogenic amino acids. The central liver illustration highlights the integration of these pathways in the regulation of net hepatic glucose production and maintenance of euglycemia. Color coding: pink-shaded elements indicate glucose-consuming pathways (glycogenesis and glycolysis); blue-shaded elements indicate glucose-producing pathways (glycogenolysis and gluconeogenesis). Black arrows indicate metabolic flux. ATP adenosine triphosphate, ADP adenosine diphosphate; DHAP, dihydroxyacetone phosphate, GAP glyceraldehyde-3-phosphate, HK hexokinase, GK glucokinase, PC pyruvate carboxylase, PEP phosphoenolpyruvate, PEPCK phosphoenolpyruvate carboxykinase, FBPase fructose-1,6-bisphosphatase, G6Pase glucose-6-phosphatase, NAD+ nicotinamide adenine dinucleotide (oxidized form), NADH nicotinamide adenine dinucleotide (reduced form), Pi inorganic phosphate, UDP-glucose uridine diphosphate glucose.

Hepatic glucose production is a core determinant of glycemic stability and whole-body metabolic homeostasis. In humans, the liver provides the dominant fraction of endogenous glucose output (approximately 90%), while the kidneys contribute a smaller but measurable proportion (about 5%) in the postabsorptive state^1^. This coordinated organ-level glucose release is indispensable for maintaining circulating glucose within a physiologic range and for continuously supplying oxidizable substrate to peripheral tissues. Under normal conditions, plasma glucose remains relatively stable in both fed and fasting states because the liver functions as a dynamic glucose buffer, alternately storing and releasing glucose according to nutritional and hormonal signals. Net hepatic glucose output is therefore not a single process, but the result of integrated flux through four interconnected pathways: gluconeogenesis, glycogenolysis, glycogenesis, and glycolysis^2^ (Fig. 1).

In the fed state, hepatocytes preferentially extract glucose from portal and systemic blood and convert it into glycogen (glycogenesis). In contrast, during fasting, the liver increases glucose release via glycogenolysis and gluconeogenesis to sustain systemic glucose availability^3,4^. Glycogenesis is the enzymatic conversion of glucose into glycogen for storage, whereas glycogenolysis is the enzymatic degradation of glycogen to generate glucose equivalents for export^5^. Glycolysis is a cytosolic pathway of ten sequential enzymatic reactions that converts glucose to pyruvate with a net production of two molecules of adenosine triphosphate (ATP) per molecule of glucose^6^. Importantly, hepatic glucose production should be distinguished from hepatic energy production: the liver can generate ATP through multiple oxidative pathways while independently regulating glucose export to meet systemic demands. As fasting duration increases or energy demand rises (for example, during prolonged physical activity), hepatic glycogen stores progressively decline. Once glycogen reserves become limited, gluconeogenesis becomes the principal contributor to hepatic glucose output. Through de novo glucose synthesis from non-carbohydrate precursors—primarily lactate, glycerol, and glucogenic amino acids—the liver preserves euglycemia and secures a continuous glucose supply for glucose-dependent tissues, especially the brain and erythrocytes^7^. This metabolic transition highlights gluconeogenesis as a tightly regulated and physiologically essential adaptive program rather than a simple backup pathway^8^. Accordingly, this review focuses on the principal regulatory mechanisms that govern gluconeogenesis under physiological conditions and examines how disruption of these controls contributes to metabolic and endocrine disease states.

## Materials and methods

A comprehensive literature search was conducted across multiple databases, including MEDLINE (via PubMed), Scopus, Web of Science, and Google Scholar. The search included publications from 1978 through 2026 to capture both foundational and recent developments in the field. A total of 149 articles were selected for inclusion in this review. Most of the selected studies were published within the past 10 years, ensuring the incorporation of current evidence. The search strategy was developed using a combination of controlled vocabulary and free-text terms. Medical subject headings (MeSH) were applied for searches in PubMed, whereas MeSH and EMTREE terms were used in Scopus. Additional free-text keywords were included to maximize the retrieval of relevant studies. The search terms included “gluconeogenesis,” “glycogenolysis,” “glycolysis,” “hypoglycemia,” “insulin,” “diabetes,” “metabolic associated steatotic liver disease,” “obesity,” “glucagon-like peptide-1 receptor agonists,” “tumor metabolism,” and “carbohydrate metabolism,” as well as combinations of these terms. The review focused on studies addressing the regulation of gluconeogenesis under physiological conditions and its dysregulation in pathological states. Particular attention was given to research describing the clinical implications of altered gluconeogenesis in metabolic disorders. Only studies published in peer-reviewed journals within the defined time frame were considered. No restrictions were applied regarding study design, with the exception of conference abstracts, which were excluded. Eligible studies were required to report clearly defined outcomes relevant to hepatic glucose metabolism and its regulation. This review is based exclusively on previously published studies and does not include new research involving human participants or animals. A narrative synthesis approach was used to analyze and summarize the available evidence. As a nonsystematic narrative review, the analysis may be subject to selection bias.

## Regulatory mechanisms of gluconeogenesis

### Physiological control of glucose homeostasis

**Fig. 2:**
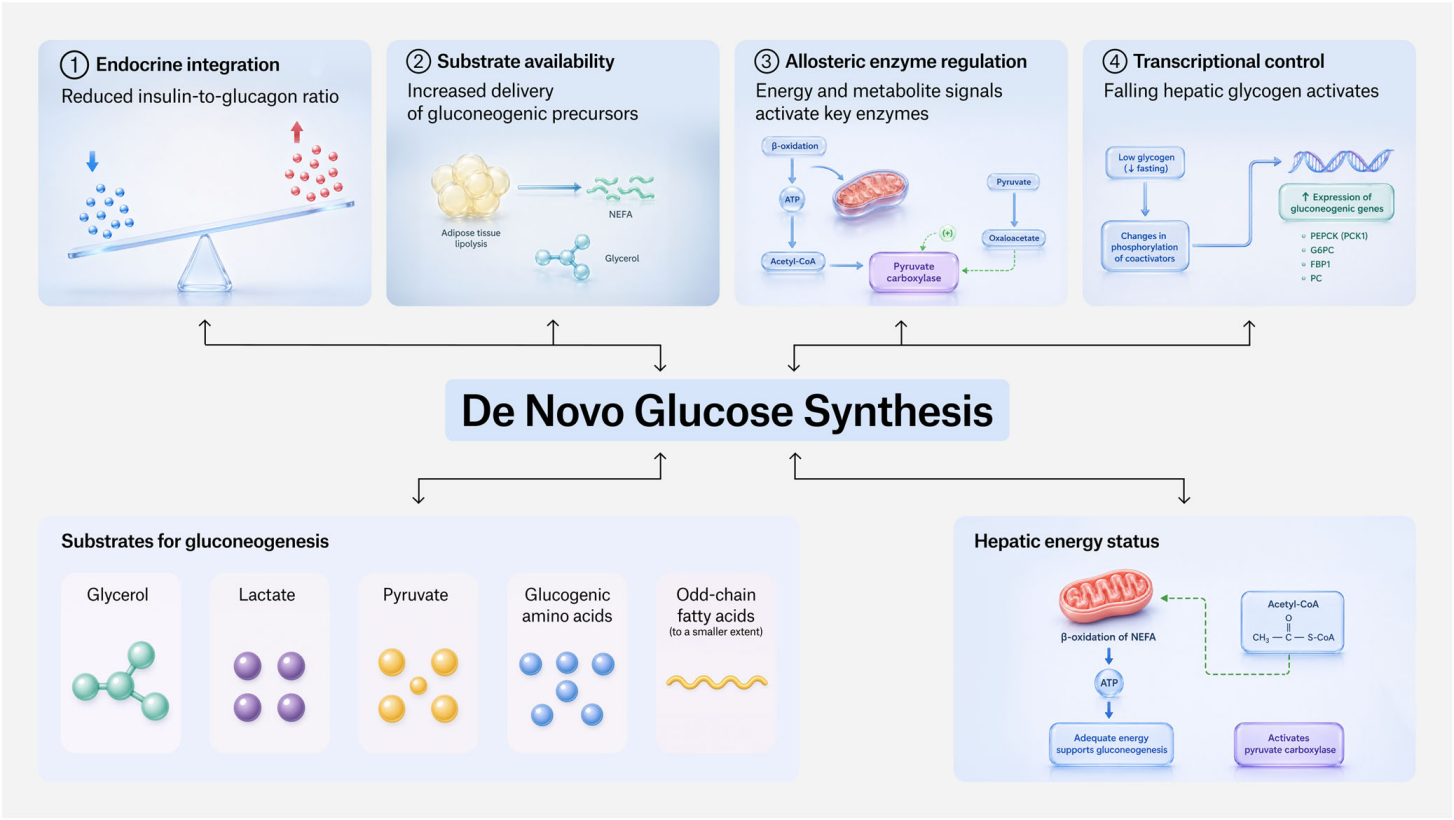
Physiological regulation of fasting-induced de novo glucose synthesis. The figure summarizes the major mechanisms regulating hepatic gluconeogenesis during fasting. A reduced insulin-to-glucagon ratio stimulates hepatic glucose production, while increased availability of gluconeogenic substrates supports glucose synthesis. Mitochondrial β-oxidation generates ATP and acetyl-CoA, providing energy and allosteric signals that promote gluconeogenic flux through activation of pyruvate carboxylase. During prolonged fasting, depletion of hepatic glycogen induces transcriptional programs that increase the expression of key gluconeogenic enzymes. The lower section illustrates the principal substrates contributing to gluconeogenesis (glycerol, lactate, pyruvate, glucogenic amino acids, and carbon intermediates derived from odd-chain fatty acids) and highlights the importance of hepatic energy status in sustaining glucose production. ATP adenosine triphosphate, Acetyl-CoA acetyl coenzyme A, FBP1 fructose-1,6-bisphosphatase 1, G6PC glucose-6-phosphatase catalytic subunit, NEFA non-esterified fatty acids, PC pyruvate carboxylase, PCK1 phosphoenolpyruvate carboxykinase 1, PEPCK phosphoenolpyruvate carboxykinase.

Maintenance of blood glucose within a narrow physiological range is a fundamental requirement for metabolic stability and organ function^9^. In clinical practice, fasting plasma glucose is routinely assessed after at least 8 h without caloric intake, typically using a morning blood sample collected before breakfast; glucose is quantified in plasma or serum by enzymatic methods or automated chemistry analyzers. In most laboratories, normal fasting values are approximately 70–100 mg/dL (3.9–5.6 mmol/L)^10^. Random blood glucose testing, in contrast, does not require fasting and provides an immediate estimate of glycemic status for real-time clinical decisions. In individuals without diabetes mellitus, random glucose concentrations are generally considered normal in the range of about 70–140 mg/dL (3.9–7.8 mmol/L)^11,12^. During progressive fasting, endogenous glucose production shifts toward de novo synthesis. Although the liver remains the dominant gluconeogenic organ, the kidney becomes increasingly important with prolonged nutrient deprivation, and renal gluconeogenesis may account for up to approximately 25% of endogenous glucose production after around 60 h of fasting^1^. As hepatic glycogen reserves decline, the proportional contribution of gluconeogenesis rises, thereby sustaining circulating glucose under conditions of reduced nutrient availability and preventing severe neuroglycopenia^7^. From a clinical perspective, hypoglycemia is diagnosed using Whipple’s triad: (1) low plasma glucose, (2) neurogenic and/or neuroglycopenic symptoms or signs, and (3) symptom resolution after correction of glucose levels. In adults without diabetes, hypoglycemia may be insulin-mediated or non-insulin-mediated. Because a single universal cutoff is not adequate for all settings, pragmatic thresholds are used: <3.9 mmol/L (70 mg/dL) is an alert value requiring assessment and preventive action, whereas <3.0 mmol/L (54 mg/dL) represents clinically significant hypoglycemia, where autonomic symptoms and cognitive dysfunction are more likely. Mechanistically, however, hypoglycemia should be interpreted mainly as a consequence of inadequate glucose production and/or excessive glucose utilization, rather than as the primary initiating signal for gluconeogenesis. Physiological activation of fasting gluconeogenesis is primarily governed by endocrine and metabolic integration: a reduced insulin-to-glucagon ratio, substrate availability, allosteric enzyme regulation, and transcriptional control of key gluconeogenic genes. At the biochemical level, pathway flux depends on both precursor supply and hepatic energy state (Fig. 2).

Lipolysis in white adipose tissue releases non-esterified fatty acids (NEFA) and glycerol; hepatic beta-oxidation of NEFA generates ATP and mitochondrial acetyl–coenzyme A (acetyl-CoA). Acetyl-CoA allosterically activates pyruvate carboxylase, promoting conversion of pyruvate to oxaloacetate and increasing gluconeogenic throughput. Glycerol enters the pathway after phosphorylation and conversion to dihydroxyacetone phosphate (DHAP), while lactate, pyruvate, glucogenic amino acids, and to a smaller extent carbons derived from odd-chain fatty acids also contribute to glucose synthesis. In parallel, falling hepatic glycogen during fasting alters phosphorylation-dependent regulation of transcriptional coactivators, which amplifies expression of gluconeogenic enzymes and reinforces hepatic glucose output^2,13–18^.

### Key regulatory enzymes and inborn errors

Gluconeogenesis is initiated in the mitochondrion, where pyruvate undergoes ATP-dependent carboxylation by pyruvate carboxylase to generate oxaloacetate^19^. Because oxaloacetate cannot efficiently traverse the inner mitochondrial membrane, mitochondrial carbon is exported indirectly: oxaloacetate is first converted to malate or aspartate, transported to the cytosol, and then reoxidized or transaminated back to oxaloacetate^20,21^. Oxaloacetate is subsequently converted to phosphoenolpyruvate by phosphoenolpyruvate carboxykinase, using guanosine triphosphate; depending on species and physiological context, this step may occur predominantly in the cytosol or partly in mitochondria, thereby coupling mitochondrial substrate flux to cytosolic glucose synthesis^22^. From phosphoenolpyruvate onward, most reactions proceed through reversal of near-equilibrium glycolytic steps until fructose-1,6-bisphosphate is formed. At this point, gluconeogenesis bypasses an irreversible glycolytic reaction through fructose-1,6-bisphosphatase, which hydrolyzes fructose-1,6-bisphosphate to fructose-6-phosphate and serves as a major regulatory checkpoint of pathway flux^23^. After isomerization to glucose-6-phosphate, the terminal gluconeogenic step occurs in the endoplasmic reticulum: glucose-6-phosphatase hydrolyzes glucose-6-phosphate to free glucose, enabling glucose release into the circulation^24^. Thus, although gluconeogenesis reuses much of the glycolytic enzymatic framework, it circumvents the three irreversible glycolytic reactions through four defining enzymes: pyruvate carboxylase, phosphoenolpyruvate carboxykinase, fructose-1,6-bisphosphatase, and glucose-6-phosphatase^25^.

Accordingly, the key irreversible transformations in gluconeogenesis are^26^:

- conversion of pyruvate to phosphoenolpyruvate via oxaloacetate (pyruvate carboxylase + phosphoenolpyruvate carboxykinase);
- hydrolytic dephosphorylation of fructose-1,6-bisphosphate (fructose-1,6-bisphosphatase);
- hydrolytic dephosphorylation of glucose-6-phosphate (glucose-6-phosphatase).

Pathogenic variants affecting these enzymes, or broader defects in pathway regulation, compromise the capacity to maintain glucose homeostasis and can produce severe multisystem disease^25^. Pyruvate carboxylase deficiency is inherited in an autosomal recessive manner and shows marked phenotypic heterogeneity, from fulminant neonatal/infantile encephalopathy to milder adult phenotypes. This broad spectrum reflects the pleiotropic role of pyruvate carboxylase, which is essential not only for gluconeogenesis but also participates in lipogenesis and neurotransmitter biosynthesis. Three major clinical forms are recognized^27,28^:

- *Type A (infantile form):* typically presents in infancy with recurrent metabolic decompensation, lactic acidosis, psychomotor delay, growth failure, hypotonia, pyramidal and extrapyramidal signs, ataxia, and seizures; structural brain abnormalities may occur, and prognosis is poor with frequent early-childhood mortality.
- *Type B (severe neonatal form):* manifests in the neonatal period with profound metabolic instability, including hypothermia, respiratory insufficiency, severe lactic acidosis, hyperammonemia, frequent hypoglycemia, and severe neurologic impairment; death often occurs within the first months of life (commonly by 8 months).
- *Type C (intermittent/attenuated form):* characterized by a comparatively milder course, with normal or only mildly delayed neurodevelopment, motor/gait abnormalities, intermittent seizures or movement disorders, episodic metabolic acidosis, and possible survival into adulthood.

Cytosolic phosphoenolpyruvate carboxykinase (PEPCK-C), encoded by the PCK1 gene, catalyzes a key rate-controlling step of gluconeogenesis by converting oxaloacetate to phosphoenolpyruvate in the cytosol. A second isoform, mitochondrial phosphoenolpyruvate carboxykinase (PEPCK-M), is encoded separately and is not equivalently affected in disorders specifically involving PCK1. Deficiency of PCK1 is an ultra-rare inborn error of metabolism characterized by fasting hypoglycemia, lactic acidosis, and urinary excretion of tricarboxylic acid cycle intermediates, especially fumarate. Fewer than approximately 30 cases have been reported to date, with pathogenic variants predominantly missense or splice-site changes, and the full phenotypic spectrum remains incompletely defined^29,30^. Fructose-1,6-bisphosphatase (FBPase) deficiency, caused by pathogenic variants in FBP1, is an autosomal recessive gluconeogenic disorder classically presenting with hypoglycemia and lactic acidosis. In addition to recurrent hypoglycemic episodes, affected patients may exhibit hepatomegaly, fever, respiratory distress, and seizures. Because its presentation overlaps with other disorders of intermediary energy metabolism, FBPase deficiency is frequently underrecognized. Early identification of the characteristic triad—hypoglycemia, ketosis, and metabolic acidosis—is therefore clinically critical for reducing morbidity. Age-dependent physiology explains the early onset: gluconeogenesis is recruited sooner in neonates and young children, whose glycogen reserves are limited relative to metabolic demand, so many patients manifest disease from infancy and sometimes from the first days of life. During infancy and toddlerhood, severe metabolic decompensation can progress to seizures, neurological injury, and life-threatening events; by contrast, acute episodes are less frequent in adults, in part because larger glycogen stores provide greater buffering capacity^31–34^. Glucose-6-phosphatase alpha (G6Pase-α), encoded by G6PC, catalyzes hydrolysis of glucose-6-phosphate to free glucose and inorganic phosphate, the terminal reaction of gluconeogenesis and glycogenolysis. Functionally, this enzyme occupies a central metabolic junction connecting glycolysis, glycogen synthesis, the pentose phosphate pathway, the hexosamine biosynthetic pathway, and hepatic glucose release. For this reason, G6Pase-α is often considered a gatekeeper of endogenous glucose production between meals. Pathogenic variants in G6PC (chromosomal locus 17q21) cause glycogen storage disease type Ia (GSD-Ia), an autosomal recessive disorder with profound disturbances in glucose homeostasis. Typical manifestations include fasting hypoglycemia, hepatomegaly, nephromegaly, hyperlipidemia, hyperuricemia, lactic acidemia, and growth retardation^24,35^. Taken together, these inherited disorders demonstrate that gluconeogenic enzymes are indispensable for carbohydrate homeostasis. Several enzymes—including pyruvate carboxylase and fructose-1,6-bisphosphatase—act as direct drivers of gluconeogenic flux and therefore represent core control nodes in glucose production^23,36^. Beyond its role in maintaining plasma glucose, gluconeogenesis also contributes to metabolic housekeeping by removing circulating three-carbon substrates, particularly lactate and glycerol, and reincorporating them into glucose. Importantly, pathway flux is not static; its magnitude and direction are continuously adjusted by integrated hormonal, allosteric, and substrate-dependent signals that match hepatic glucose output to systemic energy demand while minimizing futile cycling and inappropriate glucose overproduction.

### Hormonal regulation of gluconeogenesis

Glucagon is a principal coordinator of this adaptive network. It lowers hepatic fructose-2,6-bisphosphate, thereby releasing inhibition of fructose-1,6-bisphosphatase; it promotes phosphorylation-dependent inactivation of pyruvate kinase, redirecting carbon away from glycolysis toward gluconeogenesis; and through cyclic adenosine monophosphate (cAMP)/cAMP response element-mediated transcriptional programs, it upregulates key enzymes such as phosphoenolpyruvate carboxykinase. In parallel, mitochondrial acetyl–coenzyme A (acetyl-CoA)—generated predominantly by fatty acid beta-oxidation—acts as an allosteric activator of pyruvate carboxylase, whereas adenosine monophosphate (AMP) allosterically inhibits fructose-1,6-bisphosphatase, coupling pathway activity to cellular energy charge. Finally, changes in precursor supply (including lactate, glycerol, and amino acids) provide an additional regulatory layer by constraining or amplifying pathway throughput. Collectively, these mechanisms define a multilayered control architecture that aligns gluconeogenic flux with physiological need and, through allosteric and endocrine integration, helps prevent excessive glucose synthesis and metabolic conflict^37–41^.

From an endocrine standpoint, gluconeogenesis is regulated by a coordinated hormonal network in which glucagon and glucocorticoids are dominant stimulatory signals, while insulin provides the principal inhibitory counterbalance. Glucagon, secreted by pancreatic alpha cells, promotes hepatic glucose production by increasing transcription of key gluconeogenic enzymes, especially phosphoenolpyruvate carboxykinase and glucose-6-phosphatase, which represent major control points in pathway flux^42^. In parallel with its hepatic actions, glucagon reorganizes interorgan substrate traffic to secure carbon and energy for glucose synthesis. It exerts a catabolic influence on skeletal muscle amino acid economy, increasing net delivery of amino acids to the splanchnic compartment. Regional tracer kinetics together with increased 3-methylhistidine release indicate that essential amino acid efflux under high glucagon exposure is driven primarily by suppression of muscle protein synthesis rather than by a dominant increase in proteolysis. Elevated arterial alpha-aminoadipic acid further supports intensified lysine catabolism in the splanchnic bed during glucagon stimulation. At the signaling level in muscle, glucagon activates adenosine monophosphate-activated protein kinase, suppresses mechanistic target of rapamycin complex 1, and promotes unc-51-like kinase 1 phosphorylation, thereby favoring autophagy and protein turnover. Increased L-leucine oxidation also weakens anabolic target of rapamycin signaling, reinforcing inhibition of muscle protein synthesis. In the liver, glucagon receptor signaling through the cyclic adenosine monophosphate–protein kinase A–cAMP response element-binding protein axis upregulates amino acid transporters (for example, SLC7A2, SLC25A15, SLC38A5), enhancing hepatocellular amino acid uptake and supporting gluconeogenic substrate supply^43–45^. Glucagon additionally stimulates hepatic fatty acid oxidation, thereby supplying ATP and reducing equivalents required to sustain gluconeogenic flux^46^. Glucocorticoids add a second major layer of pro-gluconeogenic control. In hepatocytes, they stimulate glucose production, whereas in skeletal muscle and white adipose tissue they reduce insulin-mediated glucose uptake and utilization, thus favoring hyperglycemia and insulin resistance when exposure is excessive. They also influence islet biology by modulating alpha- and beta-cell function, with downstream effects on glucagon dynamics. A central adaptive function of glucocorticoids during stress is preservation of circulating glucose for obligate glucose users, particularly the brain, via transient elevation of blood glucose. Mechanistically, glucocorticoids bind the intracellular glucocorticoid receptor to form a hormone–receptor complex that engages glucocorticoid response elements in promoters/enhancers of gluconeogenic genes, including phosphoenolpyruvate carboxykinase and the glucose-6-phosphatase catalytic subunit^47^. Beyond glucose-6-phosphatase catalytic subunit induction, glucocorticoid receptor signaling increases expression of multiple gluconeogenic nodes (including phosphoenolpyruvate carboxykinase 1) and cooperates with fasting transcriptional circuits involving forkhead box O1, peroxisome proliferator-activated receptor gamma coactivator-1 alpha, hepatocyte nuclear factor 4 alpha, and CCAAT/enhancer-binding protein beta^48–50^. These integrated transcriptional programs augment expression of rate-limiting gluconeogenic enzymes and increase hepatic glucose output, thereby raising circulating glucose^51^. A key concept is hormone synergy rather than isolated hormone action. Glucocorticoids and glucagon jointly produce a stronger pro-gluconeogenic transcriptional state than either signal alone. This cooperative effect is explained in part by enhancer “assisted loading”: glucagon-activated cAMP response element-binding protein primes/activates enhancer elements, facilitating subsequent glucocorticoid receptor binding after glucocorticoid stimulation. Importantly, glucagon activates not only single enhancers but also enhancer clusters, extending glucocorticoid receptor recruitment across clustered regulatory units and amplifying gene induction^52^. Glucocorticoids also increase extrahepatic substrate delivery by stimulating adipose lipolysis and muscle protein breakdown, increasing glycerol and amino acid availability to the liver; concurrent enhancement of fatty acid oxidation further supports gluconeogenesis energetically^53^. Thus, glucocorticoids amplify hepatic glucose production through both direct transcriptional effects and systemic substrate redistribution. Epinephrine (adrenaline) contributes prominently during acute stress and heightened energy demand. Via beta-adrenergic receptors on hepatocytes, epinephrine activates cyclic adenosine monophosphate–protein kinase A signaling, which increases transcription of gluconeogenic genes and promotes hepatic glucose production^54,55^. It also supports glucose output indirectly by stimulating glycogenolysis and by increasing peripheral release of gluconeogenic precursors, including lactate and glycerol^56–58^.

**Fig. 3:**
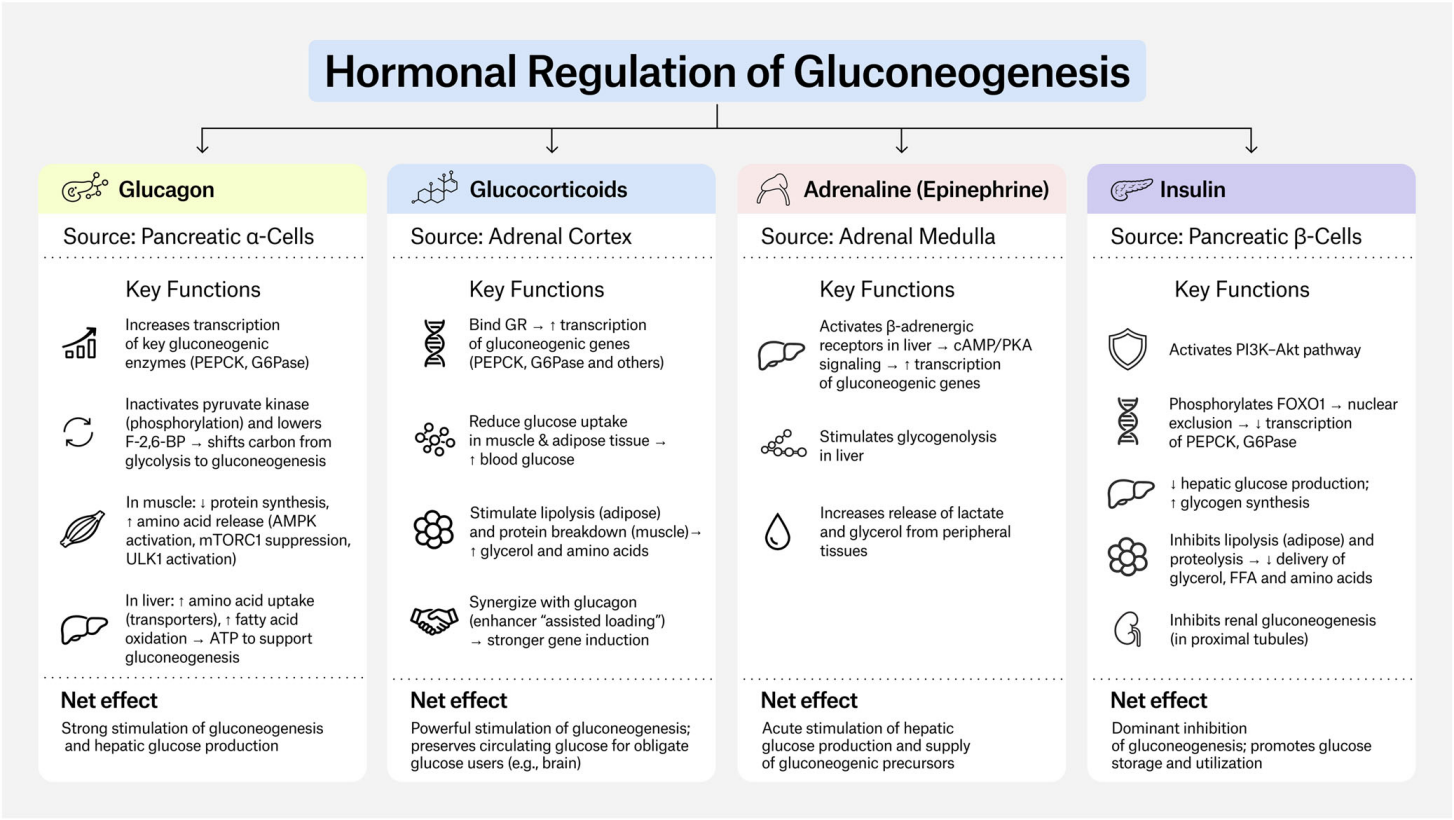
Hormonal regulation of gluconeogenesis. The figure summarizes the major hormonal regulators of hepatic gluconeogenesis and their mechanisms of action on systemic glucose homeostasis. Glucagon, secreted by pancreatic α-cells, stimulates gluconeogenesis by increasing the transcription of key gluconeogenic enzymes, inhibiting pyruvate kinase activity, and promoting amino acid uptake and fatty acid oxidation in the liver. Glucocorticoids, produced by the adrenal cortex, enhance the expression of gluconeogenic genes, reduce peripheral glucose utilization, stimulate lipolysis and proteolysis, and act synergistically with glucagon to amplify hepatic glucose production. Adrenaline (epinephrine), released from the adrenal medulla, activates β-adrenergic signaling pathways, promotes glycogenolysis, and increases the availability of gluconeogenic substrates derived from peripheral tissues. In contrast, insulin, secreted by pancreatic β-cells, suppresses gluconeogenesis through activation of the phosphoinositide 3-kinase–protein kinase B signaling pathway, inhibition of forkhead box protein O1–mediated transcription, reduction of lipolysis and proteolysis, stimulation of glycogen synthesis, and suppression of hepatic glucose production. Symbols: upward arrows indicate stimulation or increased activity; downward arrows indicate inhibition or reduced activity. Akt protein kinase B, AMPK AMP-activated protein kinase, cAMP cyclic adenosine monophosphate, F-2,6-BP fructose-2,6-bisphosphate, FFA free fatty acids, FOXO1 forkhead box protein O1, G6Pase glucose-6-phosphatase, GR glucocorticoid receptor, mTORC1 mechanistic target of rapamycin complex 1, PEPCK phosphoenolpyruvate carboxykinase, PI3K phosphoinositide 3-kinase, PKA protein kinase A, ULK1 Unc-51-like kinase 1.

Insulin functions as the dominant anti-gluconeogenic hormone in the fed state and in postprandial recovery. It suppresses hepatic glucose production, promotes glycogen synthesis, inhibits glucagon secretion from pancreatic alpha cells, suppresses adipose lipolysis, and promotes glucose uptake in skeletal muscle^9^. At the molecular level, insulin receptor activation triggers phosphatidylinositol-3-kinase–protein kinase B signaling, a central pathway for repression of hepatic gluconeogenesis^59,60^. Activated protein kinase B phosphorylates forkhead box O1, causing its nuclear exclusion and decreasing transcription of major gluconeogenic genes, including phosphoenolpyruvate carboxykinase 1 and glucose-6-phosphatase catalytic subunit. Because forkhead box O1 functionally cooperates with peroxisome proliferator-activated receptor gamma coactivator-1 alpha to induce gluconeogenic gene expression, its inhibition reduces hepatic glucose output; conversely, genetic disruption of forkhead box O1 lowers glucose by limiting hepatic gluconeogenesis^61^. Insulin-mediated repression of forkhead box O1 is also linked to selective suppression of glucose-6-phosphatase catalytic subunit expression, increased hepatic glucose utilization, and reduction in blood glucose^62^. In parallel, insulin attenuates fasting transcriptional amplification by suppressing coactivators such as peroxisome proliferator-activated receptor gamma coactivator-1 alpha and cAMP response element-binding protein regulated transcription coactivator 2^63–65^. Renal gluconeogenesis is also insulin-sensitive. Proximal tubular cells represent a major gluconeogenic compartment in the kidney, and experimental studies show that insulin suppresses renal glucose output. Insulin receptor deletion in proximal tubules promotes hyperglycemia, supporting a direct inhibitory role of insulin in this nephron segment. Mechanistic data indicate suppression through insulin receptor substrate 1/protein kinase B2/mechanistic target of rapamycin complex 1 and 2 signaling, with reduced gluconeogenic enzyme activity and glucose production in isolated proximal tubules^66^. Beyond direct hepatic and renal actions, insulin lowers substrate-driven gluconeogenic flux indirectly by inhibiting adipose lipolysis (thereby reducing delivery of non-esterified fatty acids and glycerol to the liver) and by limiting amino acid release from proteolysis^62,67,68^. Overall, physiologic glucose homeostasis across feeding, fasting, and stress is maintained by dynamic integration of glucagon, glucocorticoids, catecholamines, and insulin, acting through intersecting transcriptional, allosteric, substrate-availability, and interorgan signaling mechanisms rather than through any single linear pathway (Fig. 3).

## Importance of maintaining stable glucose levels

### Adaptive metabolic transitions during fasting and tissue-specific glucose requirements

In the early phase of fasting, hepatic glycogenolysis is the dominant contributor to circulating glucose. With increasing fasting duration, however, endogenous glucose production progressively shifts toward gluconeogenesis. Quantitative studies show that gluconeogenesis contributes approximately 54% of total glucose output after 14 h of fasting, about 64% after 22 h, and up to 84% after 42 h, underscoring a time-dependent transition from glycogen-derived to newly synthesized glucose^69^. This transition is physiologically enabled by the presence of glucose-6-phosphatase in both liver and kidney, which allows these organs to release free glucose into the bloodstream and maintain systemic glucose availability during nutrient deprivation^70^. Because glucose is the principal metabolic fuel in humans, blood glucose must be maintained within a narrow physiological range. This requirement is especially critical for tissues with high or obligatory glucose demand. Central nervous system glucose sensing is highly developed, and neuronal function remains strongly glucose dependent; the brain is among the most metabolically active organs and requires continuous substrate delivery to sustain cognition, memory processing, and motor control^71,72^. In addition, the renal medulla and erythrocytes (red blood cells) depend heavily on glucose, with limited capacity to substitute alternative fuels for prolonged periods^7^. Consequently, significant hypoglycemia may cause structural and functional injury, particularly in the brain and kidney, because these tissues are highly vulnerable to energy depletion^73–76^.

### Gluconeogenesis under stress and critical illness

Maintenance of euglycemia is also essential during prolonged physical exertion. Endurance exercise markedly increases skeletal muscle glucose uptake, potentially challenging glucose availability for other organs. Despite this competition, blood glucose is usually preserved through coordinated increases in hepatic glycogenolysis and de novo glucose synthesis. At the onset of moderate- to high-intensity exercise, hepatic glucose output is driven predominantly by glycogenolysis, largely via glucagon- and noradrenaline-mediated activation of hepatic glycogen phosphorylase. During this phase, gluconeogenesis typically contributes a smaller fraction (about 10–20%) of total glucose production, with tracer studies at approximately 70% of maximal oxygen consumption (VO2max) showing a contribution near 20%. As exercise duration extends over several hours and liver glycogen becomes progressively depleted, the relative contribution of gluconeogenesis rises, approaching about 50% of hepatic glucose production in parallel with increased precursor supply to the liver. Notably, in athletes adapted to low-carbohydrate, high-fat diets, gluconeogenesis does not appear to increase above mixed-diet levels; instead, total glucose production and glycogen breakdown are lower, while gluconeogenic rates remain broadly similar. These findings suggest that adaptation to prolonged exercise reflects a broader shift in whole-body substrate partitioning rather than a major upscaling of gluconeogenic capacity itself^77–79^. Psychological and physiological stress are additional powerful modulators of glucose homeostasis. Stress-induced glucocorticoids upregulate genes involved in carbohydrate and lipid metabolism, substantially altering hepatic metabolic programs and promoting gluconeogenesis to secure energy availability during stress responses^80^. In contrast to the typically transient metabolic effects of acute stress or exercise, prolonged fasting induces sustained activation of gluconeogenesis, requiring persistent upregulation of key rate-limiting gluconeogenic enzymes^81^. Gluconeogenesis is further intensified in critical illness and other life-threatening states. During recurrent hypoglycemia, increased expression of fructose-1,6-bisphosphatase has been documented, together with decreased lactate concentrations, while glycogen-related pathways are relatively unchanged. These findings support the concept that augmented gluconeogenesis is a central protective response against severe glucose decline, including during intensive insulin therapy^82^. Sepsis similarly disrupts global metabolic homeostasis and profoundly alters carbohydrate metabolism^83^. As hepatic glycogen stores are exhausted in sepsis, gluconeogenesis becomes increasingly important for sustaining glucose output. Interestingly, gluconeogenesis from alanine, lactate, and pyruvate may decline in both early and late sepsis, whereas glycerol remains a major effective substrate^84^. Although gluconeogenesis is energetically costly, it supports continuous ATP generation, which is essential for survival in critically ill patients^85^. At the same time, excessive gluconeogenic drive can contribute to stress hyperglycemia, a state associated with adverse outcomes^86^. Taken together, these data identify gluconeogenesis as a crucial adaptive mechanism that preserves survival during fasting, prolonged exertion, stress, and critical illness, while also illustrating that dysregulated activation may become pathologic.

## Consequences of dysregulation of gluconeogenesis

Gluconeogenesis is an indispensable adaptive pathway that preserves glucose supply during fasting and metabolic stress, yet pathological activation or suppression of this pathway contributes directly to several major disease states, including chronic liver disease, diabetes mellitus, cancer cachexia, and obesity. In stress-associated hyperglycemia, exaggerated glucagon signaling is a central driver, which is why glucagon receptor antagonism has been proposed as a therapeutic strategy in acute stress settings such as burns, trauma, surgery, and critical illness^87^.

### Genetic defects of gluconeogenesis

Disruption of gluconeogenic control causes profound derangements in carbohydrate metabolism. Loss-of-function defects in enzymes responsible for irreversible reactions of de novo glucose synthesis reduce the capacity to maintain fasting glycemia and predispose to lactic acidosis. In particular, deficiencies affecting phosphoenolpyruvate carboxykinase and pyruvate carboxylase are associated with severe clinical phenotypes, including potentially fatal outcomes^88,89^. These observations underscore that intact gluconeogenic flux is not merely supportive but essential for metabolic survival under fasting conditions.

### Metabolic dysfunction-associated steatotic liver disease (MASLD)

**Fig. 4:**
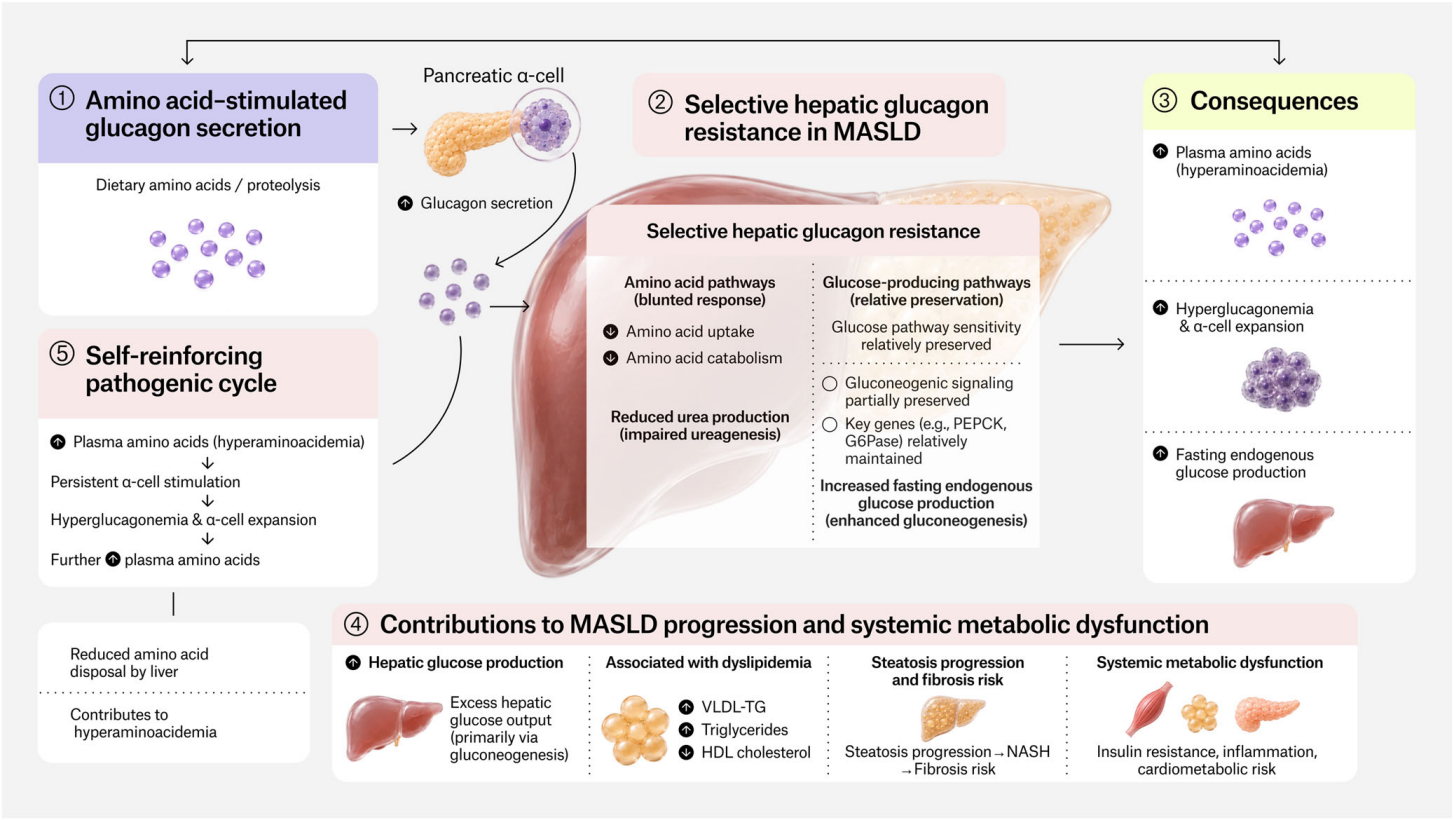
Selective hepatic glucagon resistance and disruption of the liver–alpha cell axis in metabolic dysfunction-associated steatotic liver disease (MASLD). The figure illustrates the liver–alpha cell feedback loop and its disruption in MASLD. Under normal conditions, amino acids stimulate glucagon secretion from pancreatic α-cells. Glucagon promotes hepatic amino acid uptake, amino acid catabolism, and ureagenesis, thereby maintaining amino acid homeostasis and completing the liver–alpha cell feedback axis. In MASLD, hepatic glucagon signaling becomes selectively impaired in pathways regulating amino acid metabolism, whereas glucose-producing pathways remain relatively preserved. Reduced hepatic amino acid disposal results in hyperaminoacidemia, which chronically stimulates pancreatic α-cells and promotes hyperglucagonemia and α-cell expansion. Persistent elevation of glucagon contributes to increased endogenous glucose production, dyslipidemia, hepatic steatosis progression, and systemic metabolic dysfunction. The figure summarizes the proposed mechanisms linking selective hepatic glucagon resistance to disruption of amino acid homeostasis and the development of a self-perpetuating pathogenic cycle in MASLD. Symbols: arrows indicate the direction of metabolic flux, or feedback regulation. Colored elements are used to distinguish pathological processes associated with MASLD. α-cell pancreatic alpha cell, MASLD metabolic dysfunction-associated steatotic liver disease, PEPCK phosphoenolpyruvate carboxykinase, G6Pase glucose-6-phosphatase, VLDL-TG very-low-density lipoprotein triglycerides, HDL high-density lipoprotein cholesterol, NASH nonalcoholic steatohepatitis.

In metabolic dysfunction-associated steatotic liver disease (MASLD), dysregulated glucagon action contributes to abnormal gluconeogenic regulation, especially when hepatic glycogen reserves are reduced. A key conceptual framework is the liver–alpha cell axis, which under physiological conditions links amino acid-stimulated pancreatic alpha-cell glucagon secretion to hepatic amino acid uptake, amino acid catabolism, and ureagenesis. This creates a feedback loop that stabilizes amino acid and glucagon homeostasis. In metabolically impaired states, including nonalcoholic fatty liver disease and type 2 diabetes mellitus, this axis becomes uncoupled: hepatic glucagon signaling in amino acid pathways is blunted, while glucagon effects on glucose pathways may persist. As a result, amino acid disposal and ureagenesis decline, plasma amino acids rise (hyperaminoacidemia), and persistent amino acid stimulation drives hyperglucagonemia and alpha-cell expansion, creating a self-reinforcing pathogenic cycle. In parallel, altered glucagon signaling in MASLD is linked to changes in hepatic glucose production, dyslipidemia, and intrahepatic lipid accumulation, thereby accelerating systemic metabolic dysfunction (Fig. 4).

An important unresolved issue is whether elevated glucagon in liver disease reflects impaired clearance versus impaired signaling. Human kinetic data indicate reduced glucagon clearance and prolonged glucagon half-life in stage 4 chronic kidney disease, whereas cirrhosis shows clearance parameters similar to controls^90–92^. These findings support a major renal role in glucagon elimination and suggest that, in MASLD, hyperglucagonemia is more strongly explained by glucagon resistance/signaling defects than by reduced hepatic clearance. Collectively, this supports continued evaluation of glucagon-targeted therapies in MASLD and metabolic dysfunction-associated steatohepatitis.

### Diabetes mellitus and hyperglycemia

In diabetes mellitus, excessive glucagon signaling and increased hepatic gluconeogenic flux are major determinants of fasting and postabsorptive hyperglycemia. Although diabetes has classically been framed through insulin deficiency and/or insulin resistance, growing evidence indicates that hyperglucagonemia and disordered glucagon regulation are equally central in both type 1 and type 2 diabetes. Under normal physiology, insulin suppresses glucagon secretion; in diabetes, this inhibitory cross-talk is impaired, permitting persistent glucagon action despite hyperglycemia. In pancreatic beta cells, glucose-stimulated insulin secretion involves adenylate cyclase, cyclic adenosine monophosphate, and protein kinase A signaling. In alpha cells, low glucose activates multiple G protein-coupled receptor pathways that converge on adenylate cyclase/cyclic adenosine monophosphate/protein kinase A and cAMP response element-binding protein-dependent glucagon gene regulation. Additional receptor signaling through phospholipase C, inositol trisphosphate, and exchange protein directly activated by cyclic adenosine monophosphate 2 further promotes glucagon secretion. The downstream effect is sustained hepatic glucose output via multiple gluconeogenic mechanisms. Clinically, elevated glucagon concentrations are common across diabetic populations^93–96^.

### Cancer cachexia and energy imbalance

Cancer-associated cachexia is strongly linked to increased hepatic gluconeogenesis^97,98^. Because glucose synthesis via gluconeogenesis is energetically expensive relative to glycolysis, sustained pathway activation imposes a high systemic energy burden that can contribute to progressive weight loss even when caloric intake is not profoundly reduced^99^. A comparable catabolic logic is seen in prolonged fasting, where muscle-derived amino acids become major gluconeogenic substrates^43,100^. Alanine tends to dominate earlier, whereas glutamine contribution becomes more prominent with longer fasting duration^101^. In the liver, these substrates enter via conversion to pyruvate and oxaloacetate before integration into gluconeogenic flux^102^. In cancer, this process is further amplified by tumor-associated inflammation. Cytokines such as interleukin-6 and tumor necrosis factor alpha upregulate hepatic gluconeogenic enzyme expression, while tumor metabolism increases lactate production. This intensifies Cori cycle turnover, in which lactate is recycled to glucose at substantial energetic cost to the host. In parallel, the Warburg phenotype (high lactate production despite oxygen availability) supports tumor progression and treatment resistance, reinforcing a maladaptive host–tumor metabolic coupling^103–105^.

### Obesity and substrate-driven gluconeogenesis

In obesity, excess substrate delivery is a major driver of increased gluconeogenic activity^106^. During triglyceride trafficking from intestine to adipose tissue and muscle, hydrolysis releases glycerol, which is transported to the liver and serves as an efficient gluconeogenic precursor^107,108^. Obesity and metabolic syndrome therefore show increased gluconeogenic tone, supported by expansion of metabolically active visceral adipose tissue^109–113^. Because adipose homeostasis depends on the balance of lipolysis and lipogenesis, excessive visceral lipolysis increases portal free fatty acid flux, favoring hepatic gluconeogenic activation^114^. Mechanistically, mitochondrial pyruvate carboxylase generates oxaloacetate from pyruvate, after which cytosolic phosphoenolpyruvate carboxykinase and glucose-6-phosphatase complete de novo glucose production; pyruvate carboxylase activity is often increased in obesity^115^. A second major mechanism is hepatic insulin resistance. Hyperinsulinemic-euglycemic clamp studies demonstrate that gluconeogenesis remains inappropriately elevated in obesity relative to lean controls, indicating failure of normal insulin-mediated suppression of endogenous glucose production. Elevated plasma free fatty acids further augment this process^116^. Importantly, increased gluconeogenesis is not only a glycemic defect but also an energetic one: clinical data indicate strong association between gluconeogenic activity and resting energy expenditure, with gluconeogenesis accounting for a substantial fraction of total resting energy expenditure in insulin-resistant individuals^117^.

### Pharmacological modulation of gluconeogenesis

Because excessive hepatic glucose production is central to dysglycemia, gluconeogenesis is a major therapeutic target. Metformin remains foundational in type 2 diabetes mellitus, obesity-associated dysglycemia, and metabolic syndrome due to suppression of hepatic glucose output and improvement in hepatic insulin sensitivity^118^. Glucagon-like peptide-1 receptor agonists are widely used for glycemic control and weight reduction^119^. Their metabolic benefits include delayed gastric emptying, increased satiety, reduced appetite, and partial suppression of glucagon signaling, thereby lowering hepatic glucose production^120^. This is especially relevant in type 2 diabetes, where relative insulin deficiency and hyperglucagonemia coexist^121^. Use in people without diabetes for weight management is more recent; long-term metabolic safety and durability remain under evaluation^122^. Rising off-label cosmetic use heightens the need for strict risk–benefit assessment^123^. Some studies report hypoglycemia in specific contexts^124–127^, emphasizing that therapy selection should account for baseline metabolic status and concomitant treatments. Ongoing follow-up is needed to define long-term effects on carbohydrate metabolism, body composition, gastrointestinal function, and hepatic/renal outcomes^128^. Dual incretin agonism (glucose-dependent insulinotropic polypeptide plus glucagon-like peptide-1 receptor activation), exemplified by tirzepatide, provides additional glycemic and weight benefits. Direct receptor effects occur in the central nervous system, pancreas, and stomach, with secondary systemic effects including improved insulin sensitivity, lower hyperglycemia, and improved lipid handling. Clinical data show dose-dependent weight loss with mostly mild-to-moderate transient gastrointestinal adverse events; importantly, severe clinically significant hypoglycemia is uncommon in typical use settings. Synergistic reduction of glucagon signaling is a plausible contributor to reduced gluconeogenic drive^129^. The expanding “longevity” drug landscape has increased interest in mechanistic target of rapamycin inhibition. While intermittent or short-term rapamycin exposure can mimic some calorie-restriction-like effects, chronic inhibition is associated with insulin resistance, glucose intolerance, and increased hepatic gluconeogenesis despite reductions in adiposity. A key proposed mechanism is disruption of mechanistic target of rapamycin complex 2, which impairs insulin-mediated suppression of hepatic glucose production^130,131^. Therefore, use of metabolic drugs outside clear medical indications should be approached cautiously, with explicit risk–benefit evaluation and attention to preservation of essential adaptive glucose-regulatory pathways. Overall, dysregulated gluconeogenesis is both a marker and a driver of multisystem metabolic disease. Its consequences extend beyond hyperglycemia to include altered amino acid handling, lipid dysregulation, increased energetic burden, and maladaptive interorgan signaling.

## Challenges, knowledge gaps, and future directions

Although the fundamental enzymatic steps and regulatory pathways of gluconeogenesis have been extensively characterized, significant gaps in knowledge remain. Current evidence highlights several unresolved questions related to the quantitative contribution, regulation, and clinical relevance of gluconeogenesis in different metabolic states. One important aspect that has gained increasing attention in recent years is the spatial organization of metabolic processes within the liver. The liver preserves systemic glucose and lipid balance through tightly coordinated metabolic programs, and this coordination depends heavily on liver zonation—the spatial separation of metabolic functions across the hepatic lobule. The hepatic lobule is organized as a hexagonal structural unit in which oxygen- and nutrient-rich blood travels from portal areas at the lobular periphery toward the central vein. Along this periportal-to-pericentral gradient, hepatocytes experience different microenvironments, resulting in position-dependent gene expression and metabolic specialization. Periportal hepatocytes preferentially support gluconeogenesis, cholesterol biosynthesis, fatty acid and amino acid metabolism, oxidative phosphorylation, urea cycle activity, and aspects of drug metabolism, whereas pericentral hepatocytes are enriched for glycolysis, bile acid synthesis, xenobiotic handling, glutamine synthesis, glucose uptake, lipogenesis, and ketogenesis. These findings emphasize marked transcriptional and functional heterogeneity across the periportal–pericentral axis^112,132^. However, a major conceptual gap remains: many zonation studies implicitly treat zonal gene signatures as static, despite evidence that they are dynamically remodeled by nutritional state. During feeding, gluconeogenic gene expression is relatively low throughout the lobule. In early fasting, expression rises first in periportal hepatocytes; with prolonged fasting, pericentral hepatocytes also increase gluconeogenic gene expression and activity; and during starvation, pericentral and periportal cells can display similarly elevated gluconeogenic profiles. This dynamic reorganization indicates that zonation is condition-dependent rather than fixed and requires context-specific interpretation in physiology and disease^133^.

A second layer of complexity is circadian control. Hepatic gluconeogenesis is synchronized with environmental and behavioral cycles by the circadian clock, and at least approximately 10% of the liver transcriptome exhibits rhythmic expression. Cyclic adenosine monophosphate-responsive element-binding protein, hepatocyte-specific (CREBH)—an endoplasmic reticulum-tethered transcription factor enriched in liver—has roles in the acute-phase response and lipid regulation, and also acts as a circadian regulator of glucose homeostasis. Functional interaction between CREBH and peroxisome proliferator-activated receptor alpha (PPARα) can synergistically enhance transcriptional programs linked to hepatic gluconeogenesis^134–137^. Nevertheless, the physiological significance of combining zonation and circadian regulation for net hepatic glucose output remains insufficiently defined. Future work should quantify how spatiotemporal specialization of hepatocytes contributes to whole-body glucose homeostasis.

Control of substrate flux into gluconeogenesis is likewise distributed across multiple pathways and cannot be reduced to one dominant substrate or one dominant regulatory switch. Under physiologic fasting conditions, glycerol appears to contribute a large fraction of gluconeogenic carbon in hepatocytes, while pyruvate/lactate and glutamine contributions vary by context. Glucagon signaling can increase flux from several substrates simultaneously, with a particularly strong relative increase in glutamine-derived carbon entry. In vivo protein kinase A (PKA) activation studies also support a central role for glycerol and suggest asymmetric interconversion between lactate and glycerol pools. Even so, rules governing substrate prioritization and substrate switching remain incomplete, underscoring the need for integrated, physiology-based flux studies across tissues and hormonal states^138^.

Although glucagon, insulin, and glucocorticoids are recognized core regulators of hepatic glucose production, their integration is not yet fully resolved. Emerging evidence indicates that fasting regulation is organized as a multilayered transcriptional network. Glucocorticoid receptor (GR) signaling diverges into distinct arms: the GR–CCAAT/enhancer-binding protein beta (C/EBPβ) arm preferentially augments gluconeogenesis, whereas the GR–PPARα arm promotes a delayed ketogenic response that depends on de novo protein synthesis. These observations help explain how hormonal signals can produce both amplification and pathway switching through different secondary transcription factor networks^139^.

Upstream endocrine regulation of glucagon secretion is another major knowledge gap. New data identify famsin, a gut-derived hormone, as a stimulator of alpha-cell glucagon secretion via olfactory receptor 796 (OLFR796) in mice / olfactory receptor family 10 subfamily P member 1 (OR10P1) in humans, through endoplasmic reticulum calcium mobilization. Activation of this famsin–glucagon axis increases hepatic glucose production, whereas reduced famsin signaling decreases glucagon secretion and lowers blood glucose^140^. These findings strengthen the concept of a gut–islet–liver endocrine circuit and show that hormonal control of gluconeogenesis remains incompletely mapped.

Recent fasting studies further indicate that glucagon alone does not fully activate amino acid-driven gluconeogenesis. Instead, cooperative signaling between glucagon and glucocorticoids, mediated through cAMP response element-binding protein (CREB) and GR, is required for full induction of amino acid catabolic programs. In fasting, 31 amino acid catabolism-related genes were induced, with 15 showing synergistic induction by combined glucagon and corticosterone exposure. This transcriptional cooperation increases glucose production from amino acid substrates and reinforces the idea that integrated endocrine networks, rather than single-hormone models, govern gluconeogenesis^141^. Additional endocrine inputs may also be relevant. For example, follicle-stimulating hormone (FSH) has been reported to stimulate hepatic gluconeogenesis by increasing expression of key enzymes including phosphoenolpyruvate carboxykinase (PEPCK) and glucose-6-phosphatase (G6PC). Proposed signaling mediators include G protein-coupled receptor kinase 2 (GRK2), phosphorylation of AMP-activated protein kinase (AMPK), and cyclic adenosine monophosphate-regulated transcriptional coactivator 2 (CRTC2). These findings suggest that endocrine control extends beyond classical glucose-regulatory hormones and remains incompletely characterized^142^. Counterregulation to hypoglycemia is also organized as a distributed sensing network rather than a single sensor. Glucose-sensing cells are present in peripheral and central sites, especially the hepatoportal region, brainstem, and hypothalamus, with additional populations in ventral thalamic regions. These networks coordinate behavioral and autonomic responses: paraventricular thalamic circuits appear particularly relevant to motivated feeding, while other sensor groups contribute to classical counterregulation. Effector pathways include vagal activation of glucagon secretion, sympathetic stimulation of glucagon release, and activation of the hypothalamic–pituitary–adrenal (HPA) axis, which promotes epinephrine release and can also stimulate hepatic glucose production through cyclic adenosine monophosphate-dependent induction of gluconeogenic genes. The central sensing architecture and its dysregulation remain incompletely understood and require further mechanistic mapping^143^.

Methodological limitations remain a major barrier to progress. Gluconeogenesis depends on substrate availability, cellular redox status, enzyme activation/inhibition, and hormonal context; therefore, no single assay fully captures pathway behavior in vivo. A widely used approach quantifies incorporation of deuterium from body water into newly synthesized glucose, but methods estimating precursor contribution can underestimate true carbon input because a substantial fraction of carbon is transiently stored in glycogen before later release. Radioisotope and stable isotope tracer methods have improved quantification in humans, yet each technique relies on assumptions and carries propagated errors. Current platforms provide precise and reproducible estimates, but not always absolute values. Multi-tracer studies performed within the same individuals and analytic framework may help reconcile direct versus net carbon contributions to gluconeogenesis. Standardization of tracer protocols, modeling assumptions, reporting practices, and clinically meaningful endpoints is therefore a priority for the field^144–146^.

Finally, renal gluconeogenesis remains underrecognized despite growing evidence of its systemic importance. The kidney—especially proximal tubular cells—can contribute substantially to endogenous glucose production during fasting (up to approximately 40% in some settings), with lactate as a major substrate. Renal gluconeogenesis is regulated by insulin and intracellular glucose, and is further modulated by acidosis and stress hormones. Consequently, the kidney is an important determinant of both glucose and lactate homeostasis during stress. Yet the effects of acute kidney injury and chronic kidney disease on renal gluconeogenesis remain insufficiently studied. Available clinical and experimental data indicate that kidney injury is associated with reduced expression of gluconeogenic enzymes, impaired renal glucose output, and reduced lactate clearance, which may worsen systemic metabolic instability and prognosis^147,148^. Greater integration of hepatic and renal gluconeogenesis into disease models is therefore a key future direction.

## Conclusion

Gluconeogenesis represents a central metabolic pathway required for the maintenance of systemic glucose homeostasis. The capacity to generate glucose from non-carbohydrate substrates is essential during periods of limited carbohydrate availability and under conditions of increased metabolic demand, including prolonged exercise, sepsis, and physiological stress. Adequate glucose availability is critical for the function of glucose-dependent tissues such as the brain, erythrocytes, and renal cortex. Disturbances in gluconeogenic regulation are characteristic of multiple metabolic and endocrine disorders, highlighting this pathway as a relevant therapeutic target. At the same time, non-indicated suppression of de novo glucose production may disrupt metabolic balance and lead to unfavorable consequences. These considerations emphasize the need for careful and evidence-based approaches when targeting gluconeogenesis in clinical practice.

## Data Availability

No datasets were generated or analysed during the current study.

## References

[1] Ekberg, K. et al. Contributions by kidney and liver to glucose production in the postabsorptive state and after 60h of fasting. *Diabetes* **48**, 292–300 (1999). 10334304 10.2337/diabetes.48.2.292

[2] Petersen, M. C., Vatner, D. F. & Shulman, G. I. Regulation of hepatic glucose metabolism in health and disease. *Nat. Rev. Endocrinol.* **13**, 572–587 (2017). 28731034 10.1038/nrendo.2017.80 PMC5777172

[3] Pan, Y. et al. Time and dose selective glucose metabolism for glucose homeostasis and energy conversion in the liver. *npj Syst. Biol. Appl.* **10**, 107 (2024). 39349490 10.1038/s41540-024-00437-2 PMC11443093

[4] Wu, H. et al. PGC-1α, glucose metabolism and type 2 diabetes mellitus. *J. Endocrinol.* **229**, R99–R115 (2016). 27094040 10.1530/JOE-16-0021

[5] Patino, S. C. & Orrick, J. A. Biochemistry—glycogenolysis. in *StatPearls* (StatPearls Publishing, Treasure Island, FL, 2024). 31747227

[6] Kierans, S. J. & Taylor, C. T. Glycolysis: a multifaceted metabolic pathway and signaling hub. *J. Biol. Chem.* **300**, 107906 (2024). 39442619 10.1016/j.jbc.2024.107906 PMC11605472

[7] Melkonian, E. A., Asuka, E. & Schury, M. P. Physiology, gluconeogenesis. in *StatPearls* (StatPearls Publishing, Treasure Island, FL, 2023). 31082163

[8] Zhang, X., Yang, S., Chen, J. & Su, Z. Unraveling the regulation of hepatic gluconeogenesis. *Front. Endocrinol.* **9**, 802 (2019). 10.3389/fendo.2018.00802 PMC6353800 30733709

[9] Dimitriadis, G. D., Maratou, E., Kountouri, A., Board, M. & Lambadiari, V. Regulation of postabsorptive and postprandial glucose metabolism by insulin-dependent and insulin-independent mechanisms: an integrative approach. *Nutrients* **13**, 159 (2021). 33419065 10.3390/nu13010159 PMC7825450

[10] Kubihal, S., Goyal, A., Gupta, Y. & Khadgawat, R. Glucose measurement in body fluids: a ready reckoner for clinicians. *Diab. Metab. Syndr.* **15**, 45–53 (2021). 10.1016/j.dsx.2020.11.021 33310176

[11] Krinsley, J. S. & Preiser, J. C. Time in blood glucose range 70 to 140mg/dl >80% is strongly associated with increased survival in non-diabetic critically ill adults. *Crit. Care* **19**, 179 (2015). 25927986 10.1186/s13054-015-0908-7 PMC4446958

[12] Battelino, T. et al. Clinical targets for continuous glucose monitoring data interpretation: recommendations from the international consensus on time in range. *Diab. Care* **42**, 1593–1603 (2019). 10.2337/dci19-0028 PMC6973648 31177185

[13] Miyamoto, T. & Amrein, H. Gluconeogenesis: an ancient biochemical pathway with a new twist. *Fly* **11**, 218–223 (2017). 28121487 10.1080/19336934.2017.1283081 PMC5552273

[14] Tetrick, M. A. & Odle, J. What constitutes a gluconeogenic precursor? *J. Nutr.* **150**, 2239–2241 (2020). 32652033 10.1093/jn/nxaa166

[15] Zhang, B. et al. Hepatic glycogen directly regulates gluconeogenesis through an AMPK/CRTC2 axis in mice. *J. Clin. Invest.* **135**, e188363 (2025). 40454488 10.1172/JCI188363 PMC12126231

[16] Abraham, M. B. et al. ISPAD Clinical Practice Consensus Guidelines 2022: assessment and management of hypoglycemia in children and adolescents with diabetes. *Pediatr. Diab.* **23**, 1322–1340 (2022). 10.1111/pedi.13443 PMC10107518 36537534

[17] Warner, S. O. et al. Liver glycogen-induced enhancements in hypoglycemic counterregulation require neuroglucopenia. *Am. J. Physiol. Endocrinol. Metab.* **320**, E914–E924 (2021). 33779306 10.1152/ajpendo.00501.2020 PMC8424545

[18] Looi, E. & Lawler, H. M. Non-diabetic hypoglycemia: evaluation and management in adults. *J. Clin. Med.* **14**, 4393 (2025). 40648766 10.3390/jcm14134393 PMC12250112

[19] Holeček, M. Roles of malate and aspartate in gluconeogenesis in various physiological and pathological states. *Metabolism* **145**, 155614 (2023). 37286128 10.1016/j.metabol.2023.155614

[20] Fink, B. D. et al. Hepatic glutamic-oxaloacetic transaminase promotes mitochondrial respiration energized at complex II and alters whole body metabolism. *J. Biol. Chem.* **301**, 110261 (2025). 40409551 10.1016/j.jbc.2025.110261 PMC12212131

[21] Borst, P. The malate-aspartate shuttle (Borst cycle): How it started and developed into a major metabolic pathway. *IUBMB Life* **72**, 2241–2259 (2020). 32916028 10.1002/iub.2367 PMC7693074

[22] Montal, E. D. et al. PEPCK coordinates the regulation of central carbon metabolism to promote cancer cell growth. *Mol. Cell* **60**, 571–583 (2015). 26481663 10.1016/j.molcel.2015.09.025 PMC4656111

[23] Timson, D. J. Fructose 1,6-bisphosphatase: getting the message across. *Biosci. Rep.* **39**, BSR20190124 (2019). 30804231 10.1042/BSR20190124 PMC6400660

[24] Xia, Z. et al. Structural insights into glucose-6-phosphate recognition and hydrolysis by human G6PC1. *Proc. Natl. Acad. Sci. USA* **122**, e2418316122 (2025). 39847333 10.1073/pnas.2418316122 PMC11789071

[25] Wattanavanitchakorn, S. & Jitrapakdee, S. *Gluconeogenesis*. eLS (John Wiley & Sons, 2016). 10.1002/9780470015902.a0000627.pub3.

[26] Yip, J., Geng, X., Shen, J. & Ding, Y. Cerebral gluconeogenesis and diseases. *Front. Pharmacol.* **7**, 521 (2017). 28101056 10.3389/fphar.2016.00521 PMC5209353

[27] Tsygankova, P. et al. Expanding the genetic spectrum of pyruvate carboxylase deficiency with novel missense, deep intronic and structural variants. *Mol. Genet. Metab. Rep.* **32**, 100889 (2022). 35782291 10.1016/j.ymgmr.2022.100889 PMC9240867

[28] Duque Lasio, M. L. et al. Pyruvate carboxylase deficiency. in *GeneReviews*® (University of Washington, 2024 update). 20301764

[29] Burg, D. et al. Cytosolic PEPCK deficiency caused by a novel homozygous frameshift variant presenting as resolved hypoglycemia and acute liver failure at birth. *Mol. Genet. Metab. Rep.* **42**, 101175 (2024). 40092582 10.1016/j.ymgmr.2024.101175 PMC11910243

[30] Duś-Żuchowska, M. et al. Pathogenic potential of a PCK1 gene variant in cytosolic PEPCK deficiency. *Am. J. Case Rep.* **25**, e943118 (2024). 38656928 10.12659/AJCR.943118 PMC11056215

[31] Ni, Q. et al. Fructose-1,6-bisphosphatase deficiency: estimation of prevalence in the Chinese population and genotype–phenotype correlation. *Front. Genet.* **15**, 1296797 (2024). 39036704 10.3389/fgene.2024.1296797 PMC11258016

[32] Elsayed, S. M. et al. Clinical and molecular characteristics of fructose-1,6-bisphosphatase deficiency in Egyptian patients. *Orphanet. J. Rare Dis.* **20**, 595 (2025). 41267090 10.1186/s13023-025-04100-9 PMC12632066

[33] Sakuma, I. et al. Genotype–biochemical phenotype correlations in fructose 1,6-bisphosphatase deficiency. *Commun. Biol.* **6**, 787 (2023). 37507476 10.1038/s42003-023-05160-y PMC10382519

[34] Xin, B. et al. Novel compound heterozygous mutations of the FBP1 gene in hypoglycemia and lactic acidosis. *Mol. Genet. Genom. Med.* **12**, e2339 (2024). 10.1002/mgg3.2339 PMC10767684 38111981

[35] Chou, J. Y. & Mansfield, B. C. Mutations in the glucose-6-phosphatase-α (G6PC) gene that cause type Ia glycogen storage disease. *Hum. Mutat.* **29**, 921–930 (2008). 18449899 10.1002/humu.20772 PMC2475600

[36] Onodera, T. et al. Endogenous renal adiponectin drives gluconeogenesis through enhancing pyruvate and fatty acid utilization. *Nat. Commun.* **14**, 6531 (2023). 37848446 10.1038/s41467-023-42188-4 PMC10582045

[37] Chourpiliadis, C. & Mohiuddin, S. S. Biochemistry, gluconeogenesis. in *StatPearls* (StatPearls Publishing, 2026). 31335066

[38] She, F. et al. Allosteric regulation of pyruvate kinase enables efficient and robust gluconeogenesis by preventing metabolic conflicts and carbon overflow. *mSystems* **10**, e01131-24 (2025). 39873491 10.1128/msystems.01131-24 PMC11834443

[39] Wang, Y., Kwon, H., Su, X. & Wondisford, F. E. Glycerol is the major net carbon source for gluconeogenesis in mice during fasting. *Mol. Metab.* **31**, 36–44 (2020). 31918920 10.1016/j.molmet.2019.11.005 PMC6881678

[40] Shah, A. et al. Glycerol’s contribution to lactate production outside a glucose intermediate in fasting humans. *Metabolism* **132**, 155214 (2022). 35562085 10.1016/j.metabol.2022.155214

[41] Shah, A., Wang, Y. & Wondisford, F. E. Differential metabolism of glycerol based on oral versus intravenous administration in humans. *Metabolites* **12**, 890 (2022). 36295792 10.3390/metabo12100890 PMC9611849

[42] Barthel, A. & Schmoll, D. Novel concepts in insulin regulation of hepatic gluconeogenesis. *Am. J. Physiol. Endocrinol. Metab.* **285**, E685–E692 (2003). 12959935 10.1152/ajpendo.00253.2003

[43] James, H. et al. The effect of glucagon on protein catabolism during insulin deficiency: exchange of amino acids across skeletal muscle and the splanchnic bed. *Diabetes* **71**, 1636–1648 (2022). 35621914 10.2337/db22-0079 PMC9490357

[44] Kajani, S., Laker, R. C., Ratkova, E., Will, S. & Rhodes, C. J. Hepatic glucagon action: beyond glucose mobilization. *Physiol. Rev.* **104**, 1021–1060 (2024). 38300523 10.1152/physrev.00028.2023

[45] Adeva-Andany, M. M. et al. The effects of glucagon and the target of rapamycin (TOR) on skeletal muscle protein synthesis and age-dependent sarcopenia in humans. *Clin. Nutr. ESPEN* **44**, 15–25 (2021). 34330459 10.1016/j.clnesp.2021.06.025

[46] Adeva-Andany, M. M. et al. Metabolic effects of glucagon in humans. *J. Clin. Transl. Endocrinol.* **15**, 45–53 (2018). 30619718 10.1016/j.jcte.2018.12.005 PMC6312800

[47] Kuo, T., McQueen, A., Chen, T. C. & Wang, J. C. Regulation of glucose homeostasis by glucocorticoids. *Adv. Exp. Med. Biol.* **872**, 99–126 (2015). 26215992 10.1007/978-1-4939-2895-8_5 PMC6185996

[48] Vander Kooi, B. T. et al. The glucose-6-phosphatase catalytic subunit gene promoter contains both positive and negative glucocorticoid response elements. *Mol. Endocrinol.* **19**, 3001–3022 (2005). 16037130 10.1210/me.2004-0497

[49] Chang, M. & Wang, J. C. Hepatic glucocorticoid receptor action and glucose homeostasis. *Endocr. Rev.* **47**, 52–74 (2026). 40892984 10.1210/endrev/bnaf030 PMC12795704

[50] Kokkinopoulou, I., Diakoumi, A. & Moutsatsou, P. Glucocorticoid receptor signaling in diabetes. *Int. J. Mol. Sci.* **22**, 11173 (2021). 34681832 10.3390/ijms222011173 PMC8537243

[51] Sari, D. A., Samodra, G. & Kusuma, I. Y. Molecular mechanism of glucocorticoid-induced hyperglycemia. *Pharm. Rep.* **1**, 1 (2021).

[52] Goldberg, D. et al. Hormone-controlled cooperative transcription factor binding in fasting genes. *Nucleic Acids Res.* **50**, 6123–6138 (2022). 10.1093/nar/gkac358 PMC9177981 35556130

[53] Li, J. X. & Cummins, C. L. Fresh insights into glucocorticoid-induced diabetes mellitus and new therapeutic directions. *Nat. Rev. Endocrinol.* **18**, 540–557 (2022). 35585199 10.1038/s41574-022-00683-6 PMC9116713

[54] Dibe, H. A. et al. Epinephrine responsiveness in trained mouse liver. *Physiol. Rep.* **8**, e14370 (2020). 32061187 10.14814/phy2.14370 PMC7023888

[55] Šestan, M. et al. Neuronal-ILC2 interactions regulate pancreatic glucagon and glucose homeostasis. *Science* **387**, eadi3624 (2025). 39818880 10.1126/science.adi3624

[56] Phadke, D., Beller, J. P. & Tribble, C. The disparate effects of epinephrine and norepinephrine on hyperglycemia in cardiovascular surgery. *Heart Surg. Forum* **21**, E522–E526 (2018). 30604678 10.1532/hsf.2008

[57] Rokamp, K. Z. et al. Impact of polymorphism in the β2-receptor gene on the metabolic response to epinephrine after repeated hypoglycemia. *Diabetes* **72**, 728–734 (2023). 36913730 10.2337/db22-0718

[58] Alhenc-Gelas, F. & Marre, M. Young-onset type 2 diabetes: when gluconeogenesis is overfueled and out of control. *J. Clin. Endocrinol. Metab.* **109**, e1940–e1941 (2024). 38441504 10.1210/clinem/dgae123

[59] Tang, W. et al. Influence and treatment of insulin receptor substrate/PI3K/Akt-mediated insulin resistance in diabetes mellitus. *Mol. Med. Rep.* **33**, 63 (2026). 41384289 10.3892/mmr.2025.13773 PMC12744929

[60] Edgerton, D. S. et al. Insulin’s direct hepatic effect explains the inhibition of glucose production caused by insulin secretion. *JCI Insight* **2**, e91863 (2017). 28352665 10.1172/jci.insight.91863 PMC5358484

[61] Kodani, N. & Nakae, J. Tissue-specific metabolic regulation of FOXO-binding protein: FOXO does not act alone. *Cells* **9**, 702 (2020). 32182991 10.3390/cells9030702 PMC7140670

[62] Hatting, M. et al. Insulin regulation of gluconeogenesis. *Ann. N. Y. Acad. Sci.* **1411**, 21–35 (2018). 28868790 10.1111/nyas.13435 PMC5927596

[63] Han, H. S., Kwon, Y. & Koo, S. H. Role of CRTC2 in metabolic homeostasis: key regulator of whole-body energy metabolism? *Diabetes Metab. J.* **44**, 498–508 (2020). 32174060 10.4093/dmj.2019.0200 PMC7453979

[64] Lee, J. M. et al. The SMILE transcriptional corepressor inhibits cAMP response element-binding protein (CREB)-mediated transactivation of gluconeogenic genes. *J. Biol. Chem.* **293**, 13125–13133 (2018). 29950523 10.1074/jbc.RA118.002196 PMC6109926

[65] Sharabi, K. et al. Selective chemical inhibition of PGC-1α gluconeogenic activity ameliorates type 2 diabetes. *Cell* **169**, 148–160.e15 (2017). 28340340 10.1016/j.cell.2017.03.001 PMC5398763

[66] Nakamura, M. et al. Insulin promotes sodium transport but suppresses gluconeogenesis via distinct cellular pathways in human and rat renal proximal tubules. *Kidney Int.* **97**, 316–326 (2020). 31735358 10.1016/j.kint.2019.08.021

[67] Lewis, G. F. et al. Direct and indirect control of hepatic glucose production by insulin. *Cell Metab.* **33**, 709–720 (2021). 33765416 10.1016/j.cmet.2021.03.007

[68] Najjar, S. M. et al. Regulation of insulin clearance by non-esterified fatty acids. *Biomedicines* **10**, 1899 (2022). 36009446 10.3390/biomedicines10081899 PMC9405499

[69] Chandramouli, V. et al. Quantifying gluconeogenesis during fasting. *Am. J. Physiol.* **273**, E1209–E1215 (1997). 9435538 10.1152/ajpendo.1997.273.6.E1209

[70] van Schaftingen, E. & Gerin, I. The glucose-6-phosphatase system. *Biochem. J.* **362**, 513–532 (2002). 11879177 10.1042/0264-6021:3620513 PMC1222414

[71] Choi, J. H. & Kim, M. S. Homeostatic regulation of glucose metabolism by the central nervous system. *Endocrinol. Metab.* **37**, 9–25 (2022). 10.3803/EnM.2021.1364 PMC8901968 35255598

[72] Ritter, S. Monitoring and maintenance of brain glucose supply: importance of hindbrain catecholamine neurons in this multifaceted task. in *Appetite and Food Intake: Central Control* 2nd edn (ed Harris, R. B. S.) Ch. 9 (CRC Press, Boca Raton, 2017). 28880517

[73] McCrimmon, R. J. Consequences of recurrent hypoglycaemia on brain function in diabetes. *Diabetologia* **64**, 971–977 (2021). 33738528 10.1007/s00125-020-05369-0 PMC8012314

[74] Hamid, M., Benmoh, Y. & Bourazza, A. Hypoglycemic encephalopathy with extensive brain injuries: a case report. *Radiol. Case Rep.* **18**, 4495–4498 (2023). 37868008 10.1016/j.radcr.2023.09.035 PMC10589743

[75] Yun, J. S. et al. Severe hypoglycemia and the risk of end stage renal disease in type 2 diabetes. *Sci. Rep.* **11**, 4305 (2021). 33619302 10.1038/s41598-021-82838-5 PMC7900096

[76] Shih, C. J. et al. Association of hypoglycemia with incident chronic kidney disease in patients with type 2 diabetes: a nationwide population-based study. *Medicine* **94**, e771 (2015). 25906112 10.1097/MD.0000000000000771 PMC4602688

[77] Emhoff, C. A. et al. Gluconeogenesis and hepatic glycogenolysis during exercise at the lactate threshold. *J. Appl. Physiol.* **114**, 297–306 (2013). 23239870 10.1152/japplphysiol.01202.2012 PMC8846961

[78] Suh, S. H., Paik, I. Y. & Jacobs, K. Regulation of blood glucose homeostasis during prolonged exercise. *Mol. Cells* **23**, 272–279 (2007). 17646701

[79] Webster, C. C. et al. Gluconeogenesis during endurance exercise in cyclists habituated to a long-term low carbohydrate high-fat diet. *J. Physiol.* **594**, 4389–4405 (2016). 26918583 10.1113/JP271934 PMC4967730

[80] Seal, S. V. & Turner, J. D. The ‘Jekyll and Hyde’ of gluconeogenesis: early life adversity, later life stress, and metabolic disturbances. *Int. J. Mol. Sci.* **22**, 3344 (2021). 33805856 10.3390/ijms22073344 PMC8037741

[81] Oh, K. J., Han, H. S., Kim, M. J. & Koo, S. H. Transcriptional regulators of hepatic gluconeogenesis. *Arch. Pharm. Res.* **36**, 189–200 (2013). 23361586 10.1007/s12272-013-0018-5

[82] Liu, Z. et al. Recurrent hypoglycemia increases hepatic gluconeogenesis without affecting glycogen metabolism or systemic lipolysis in rat. *Metabolism* **136**, 155310 (2022). 36063868 10.1016/j.metabol.2022.155310

[83] Wasyluk, W. & Zwolak, A. Metabolic alterations in sepsis. *J. Clin. Med.* **10**, 2412 (2021). 34072402 10.3390/jcm10112412 PMC8197843

[84] de Souza Galia, W. B. et al. Gluconeogenesis is reduced from alanine, lactate and pyruvate, but maintained from glycerol, in liver perfusion of rats with early and late sepsis. *Cell Biochem. Funct.* **39**, 754–762 (2021). 33913177 10.1002/cbf.3637

[85] Sobotka, L. & Soeters, P. Basics in clinical nutrition: metabolic response to injury and sepsis. *e-SPEN Eur. e-J. Clin. Nutr. Metab.* **4**, e1–e3 (2009).

[86] Nugent, K., Edriss, H. & Selvan, K. Hyperglycemia and outcomes in patients with sepsis. *J. Thorac. Dis.* **8**, E575–E577 (2016). 27500440 10.21037/jtd.2016.05.63 PMC4958869

[87] Harp, J. B., Yancopoulos, G. D. & Gromada, J. Glucagon orchestrates stress-induced hyperglycaemia. *Diab. Obes. Metab.* **18**, 648–653 (2016). 10.1111/dom.12668 PMC5084782 27027662

[88] van den Berghe, G. Disorders of gluconeogenesis. *J. Inherit. Metab. Dis.* **19**, 470–477 (1996). 8884571 10.1007/BF01799108

[89] Becker, J. et al. Cytosolic phosphoenolpyruvate carboxykinase deficiency: cause of hypoglycemia-induced seizure and death. *Neuropediatrics* **52**, 398–402 (2021). 33445193 10.1055/s-0040-1722685

[90] McGlone, E. R., Bloom, S. R. & Tan, T. M. Glucagon resistance and metabolic-associated steatotic liver disease: a review of the evidence. *J. Endocrinol.* **261**, e230365 (2024). 38579751 10.1530/JOE-23-0365 PMC11067060

[91] Grøndahl, M. F. G. et al. Glucagon clearance is decreased in chronic kidney disease but preserved in liver cirrhosis. *Diabetes* **73**, 1641–1647 (2024). 39052774 10.2337/db24-0305 PMC11417434

[92] Pixner, T. et al. The relationship between glucose and the liver-alpha cell axis—a systematic review. *Front. Endocrinol.* **13**, 1061682 (2023). 10.3389/fendo.2022.1061682 PMC9849557 36686477

[93] Lee, S. H., Park, H. Y., Yun, J. H. & Do, E. K. Glucagon in metabolic disease: a mini-review of emerging multi-organ roles beyond glycemic control. *Front. Endocrinol.* **16**, 1645041 (2025). 10.3389/fendo.2025.1645041 PMC12350081 40822953

[94] Lubaczeuski, C. et al. Time-dependent effects of endogenous hyperglucagonemia on glucose homeostasis and hepatic glucagon action. *JCI Insight* **8**, e162255 (2023). 37140984 10.1172/jci.insight.162255 PMC10393226

[95] Barroso, E., Jurado-Aguilar, J., Wahli, W., Palomer, X. & Vázquez-Carrera, M. Increased hepatic gluconeogenesis and type 2 diabetes mellitus. *Trends Endocrinol. Metab.* **35**, 1062–1077 (2024). 38816269 10.1016/j.tem.2024.05.006

[96] Chung, S. T. et al. Increased gluconeogenesis in youth with newly diagnosed type 2 diabetes. *Diabetologia* **58**, 596–603 (2015). 25447079 10.1007/s00125-014-3455-x PMC4323952

[97] Stein, T. P. Cachexia, gluconeogenesis and progressive weight loss in cancer patients. *J. Theor. Biol.* **73**, 51–59 (1978). 692147 10.1016/0022-5193(78)90179-0

[98] Liu, Y. et al. Hepatic gluconeogenesis and PDK3 upregulation drive cancer cachexia in flies and mice. *Nat. Metab.* **7**, 823–841 (2025). 40275022 10.1038/s42255-025-01265-2 PMC12021660

[99] Bongaerts, G. P., van Halteren, H. K., Verhagen, C. A. & Wagener, D. J. Cancer cachexia demonstrates the energetic impact of gluconeogenesis in human metabolism. *Med. Hypotheses* **67**, 1213–1222 (2006). 16797873 10.1016/j.mehy.2006.04.048

[100] Argilés, J. M., Campos, N., Lopez-Pedrosa, J. M., Rueda, R. & Rodriguez-Mañas, L. Skeletal muscle regulates metabolism via interorgan crosstalk: roles in health and disease. *J. Am. Med. Dir. Assoc.* **17**, 789–796 (2016). 27324808 10.1016/j.jamda.2016.04.019

[101] Holeček, M. Origin and roles of alanine and glutamine in gluconeogenesis in the liver, kidneys, and small intestine under physiological and pathological conditions. *Int. J. Mol. Sci.* **25**, 7037 (2024). 39000145 10.3390/ijms25137037 PMC11241752

[102] Nagana Gowda, G. A., Lusk, J. A. & Pascua, V. Intracellular pyruvate-lactate-alanine cycling detected using real-time nuclear magnetic resonance spectroscopy of live cells and isolated mitochondria. *Magn. Reson. Chem.* **62**, 84–93 (2024). 38098198 10.1002/mrc.5419 PMC10872489

[103] Aguilar-Cazares, D. et al. The systemic-level repercussions of cancer-associated inflammation mediators produced in the tumor microenvironment. *Front. Endocrinol.* **13**, 929572 (2022). 10.3389/fendo.2022.929572 PMC9441602 36072935

[104] Kocianova, E., Piatrikova, V. & Golias, T. Revisiting the Warburg effect with focus on lactate. *Cancers* **14**, 6028 (2022). 36551514 10.3390/cancers14246028 PMC9776395

[105] Kumar, S., Sahu, N., Jawaid, T., Jayasingh Chellammal, H. S. & Upadhyay, P. Dual role of lactate in human health and disease. *Front. Physiol.* **16**, 1621358 (2025). 40821941 10.3389/fphys.2025.1621358 PMC12354575

[106] Berry, M. N., Phillips, J. W., Henly, D. C. & Clark, D. G. Evidence that stimulation of gluconeogenesis by fatty acids is mediated through thermodynamic mechanisms. *FEBS Lett.* **217**, 311–314 (1987). 10.1016/0014-5793(88)80694-x 2966075

[107] Edwards, M. & Mohiuddin, S. S. Biochemistry, lipolysis. in *StatPearls* (StatPearls Publishing, Treasure Island, FL, 2023). 32809399

[108] Kalemba, K. M. et al. Glycerol induces G6pc in primary mouse hepatocytes and is the preferred substrate for gluconeogenesis both in vitro and in vivo. *J. Biol. Chem.* **294**, 18017–18028 (2019). 31645433 10.1074/jbc.RA119.011033 PMC6885632

[109] Chevalier, S., Burgess, S. C., Malloy, C. R., Gougeon, R. & Marliss, E. B. The greater contribution of gluconeogenesis to glucose production in obesity is related to increased whole-body protein catabolism. *Diabetes* **55**, 675–681 (2006). 16505230 10.2337/diabetes.55.03.06.db05-1117

[110] Gastaldelli, A. et al. Separate contribution of diabetes, total fat mass, and fat topography to glucose production, gluconeogenesis, and glycogenolysis. *J. Clin. Endocrinol. Metab.* **89**, 3914–3921 (2004). 15292327 10.1210/jc.2003-031941

[111] Kundel, V. et al. Measuring visceral adipose tissue metabolic activity in sleep apnea utilizing hybrid 18F-FDG PET/MRI: a pilot study. *Nat. Sci. Sleep.* **13**, 1943–1953 (2021). 34737662 10.2147/NSS.S327341 PMC8560175

[112] Richard, A. J., White, U., Elks, C. M. & Stephens, J. M. Adipose tissue: physiology to metabolic dysfunction. in *Endotext* (eds Feingold, K. R. et al.) (MDText.com, South Dartmouth, MA, 2020).

[113] Choi, K. E., Joung, C., Pahk, K. J., Kim, H. & Pahk, K. Metabolic activity of visceral adipose tissue is associated with age-related macular degeneration: a pilot 18F-FDG PET/CT study. *Front. Endocrinol.* **14**, 1322326 (2024). 10.3389/fendo.2023.1322326 PMC10801050 38260144

[114] Saponaro, C., Gaggini, M., Carli, F. & Gastaldelli, A. The subtle balance between lipolysis and lipogenesis: a critical point in metabolic homeostasis. *Nutrients* **7**, 9453–9474 (2015). 26580649 10.3390/nu7115475 PMC4663603

[115] Pereira, R. M. et al. Strength exercise reduces hepatic pyruvate carboxylase and gluconeogenesis in DIO mice. *J. Endocrinol.* **247**, 127–138 (2020). 32805709 10.1530/JOE-20-0193

[116] Basu, R., Chandramouli, V., Dicke, B., Landau, B. & Rizza, R. Obesity and type 2 diabetes impair insulin-induced suppression of glycogenolysis as well as gluconeogenesis. *Diabetes* **54**, 1942–1948 (2005). 15983193 10.2337/diabetes.54.7.1942

[117] Quaye, E. et al. Energy expenditure due to gluconeogenesis in pathological conditions of insulin resistance. *Am. J. Physiol. Endocrinol. Metab.* **321**, E795–E801 (2021). 34693755 10.1152/ajpendo.00281.2021 PMC8714967

[118] Song, S., Andrikopoulos, S., Filippis, C., Thorburn, A. W. & Proietto, J. Mechanism of fat-induced hepatic gluconeogenesis: effect of metformin. *Am. J. Physiol. Endocrinol. Metab.* **281**, E275–E282 (2001). 11440903 10.1152/ajpendo.2001.281.2.E275

[119] Hamed, K. et al. Glucagon-like peptide-1 (GLP-1) receptor agonists: exploring their impact on diabetes, obesity, and cardiovascular health through a comprehensive literature review. *Cureus* **16**, e68390 (2024). 39355484 10.7759/cureus.68390 PMC11444311

[120] Drucker, D. J. Mechanisms of action and therapeutic application of glucagon-like peptide-1. *Cell Metab.* **27**, 740–756 (2018). 29617641 10.1016/j.cmet.2018.03.001

[121] Lee, M. et al. Higher glucagon-to-insulin ratio is associated with elevated glycated hemoglobin levels in type 2 diabetes patients. *Korean J. Intern. Med.* **34**, 1068–1077 (2019). 28882024 10.3904/kjim.2016.233 PMC6718759

[122] Myerson, M. & Paparodis, R. D. Pharmacotherapy of weight-loss and obesity with a focus on GLP-1 receptor agonists. *J. Clin. Pharmacol.* **64**, 1204–1221 (2024). 38924121 10.1002/jcph.2487

[123] Han, S. H. et al. Public interest in the off-label use of glucagon-like peptide-1 agonists (Ozempic) for cosmetic weight loss: a Google Trends analysis. *Aesthet. Surg. J.* **44**, 60–67 (2024). 10.1093/asj/sjad211 37402640

[124] Moll, H. et al. GLP-1 receptor agonists for weight reduction in people living with obesity but without diabetes: a living benefit-harm modelling study. *eClinicalMedicine* **73**, 102661 (2024). 38846069 10.1016/j.eclinm.2024.102661 PMC11154119

[125] Rosenstock, J. et al. Beneficial effects of once-daily lixisenatide on overall and postprandial glycemic levels without significant excess of hypoglycemia in type 2 diabetes inadequately controlled on a sulfonylurea with or without metformin (GetGoal-S). *J. Diab. Complications* **28**, 386–392 (2014). 10.1016/j.jdiacomp.2014.01.012 24650952

[126] Home, P. D. et al. Efficacy and tolerability of albiglutide versus placebo or pioglitazone over 1 year in people with type 2 diabetes currently taking metformin and glimepiride: HARMONY 5. *Diab. Obes. Metab.* **17**, 179–187 (2015). 10.1111/dom.12414 25406730

[127] Htike, Z. Z. et al. Efficacy and safety of glucagon-like peptide-1 receptor agonists in type 2 diabetes: a systematic review and mixed-treatment comparison analysis. *Diab. Obes. Metab.* **19**, 524–536 (2017). 10.1111/dom.12849 27981757

[128] Bobok, N. Therapeutic strategies, consequences and prospects of glucagon-like peptide-1 agonists. *Int. J. Endocrinol.* **21**, 314–321 (2025).

[129] Yang, J. et al. Tirzepatide’s innovative applications in the management of type 2 diabetes and its future prospects in cardiovascular health. *Front. Pharmacol.* **15**, 1453825 (2024). 39263564 10.3389/fphar.2024.1453825 PMC11387164

[130] Yu, D., Zeng, X., Barzilai, D., Thor, D. & Lyu, Y. X. Bridging expectations and science: a roadmap for the future of longevity interventions. *Biogerontology* **26**, 138 (2025). 40591010 10.1007/s10522-025-10278-z PMC12213962

[131] Kezic, A., Popovic, L. & Lalic, K. mTOR inhibitor therapy and metabolic consequences: where do we stand? *Oxid. Med. Cell. Longev.* **2018**, 2640342, 10.1155/2018/2640342 (2018). 30034573 10.1155/2018/2640342 PMC6035806

[132] Cunningham, R. P. & Porat-Shliom, N. Liver zonation—revisiting old questions with new technologies. *Front. Physiol.* **12**, 732929, 10.3389/fphys.2021.732929 (2021). 34566696 10.3389/fphys.2021.732929 PMC8458816

[133] Paolini, E., Longo, M., Meroni, M. & Dongiovanni, P. Hepatic zonation in MASLD: old question, new challenge in the era of spatial omics. *Int. J. Mol. Sci.* **26**, 10701 (2025). 41226735 10.3390/ijms262110701 PMC12609896

[134] Okada, J. et al. Spatial hepatocyte plasticity of gluconeogenesis during the metabolic transitions between fed, fasted and starvation states. *Nat. Metab.* **7**, 1073–1091 (2025). 40281362 10.1038/s42255-025-01269-y PMC12767623

[135] Hunter, A. L. & Ray, D. W. Circadian clock regulation of hepatic energy metabolism regulatory circuits. *Biology* **8**, 79 (2019). 31635079 10.3390/biology8040079 PMC6956161

[136] Zhang, E. E. et al. Cryptochrome mediates circadian regulation of cAMP signaling and hepatic gluconeogenesis. *Nat. Med.* **16**, 1152–1156 (2010). 20852621 10.1038/nm.2214 PMC2952072

[137] Kim, H., Zheng, Z., Walker, P. D., Kapatos, G. & Zhang, K. CREBH maintains circadian glucose homeostasis by regulating hepatic glycogenolysis and gluconeogenesis. *Mol. Cell. Biol.* **37**, e00048–17, 10.1128/MCB.00048-17 (2017). 28461393 10.1128/MCB.00048-17 PMC5492176

[138] Xu, H. et al. Glucagon changes substrate preference in gluconeogenesis. *J. Biol. Chem.* **298**, 102708 (2022). 36402444 10.1016/j.jbc.2022.102708 PMC9747632

[139] Goldberg, D., Buchshtab, N., Charni-Natan, M. & Goldstein, I. Transcriptional cascades during fasting amplify gluconeogenesis and instigate a secondary wave of ketogenic gene transcription. *Liver Int.* **44**, 2964–2982 (2024). 39162082 10.1111/liv.16077

[140] Long, A. et al. A famsin–glucagon axis mediates glucose homeostasis. *Cell Metab.* **37**, 629–639.e6 (2025). 39706194 10.1016/j.cmet.2024.11.008

[141] Korenfeld, N. et al. Fasting hormones synergistically induce amino acid catabolism genes to promote gluconeogenesis. *Cell. Mol. Gastroenterol. Hepatol.* **12**, 1021–1036 (2021). 33957303 10.1016/j.jcmgh.2021.04.017 PMC8346669

[142] Qi, X. et al. Follicle-stimulating hormone enhances hepatic gluconeogenesis by GRK2-mediated AMPK hyperphosphorylation at Ser485 in mice. *Diabetologia* **61**, 1180–1192 (2018). 29442133 10.1007/s00125-018-4562-x

[143] Thorens, B. Neuronal regulation of glucagon secretion and gluconeogenesis. *J. Diab. Investig.* **13**, 599–607 (2022). 10.1111/jdi.13745 PMC9017634 34989155

[144] Ziari, N. & Hellerstein, M. Measurement of gluconeogenesis by ^2H_2O labeling and mass isotopomer distribution analysis. *J. Biol. Chem.* **299**, 105206 (2023). 37660907 10.1016/j.jbc.2023.105206 PMC10539955

[145] Chung, S. T., Chacko, S. K., Sunehag, A. L. & Haymond, M. W. Measurements of gluconeogenesis and glycogenolysis: a methodological review. *Diabetes* **64**, 3996–4010 (2015). 26604176 10.2337/db15-0640 PMC4657587

[146] Shah, A. M. & Wondisford, F. E. Tracking the carbons supplying gluconeogenesis. *J. Biol. Chem.* **295**, 14419–14429 (2020). 32817317 10.1074/jbc.REV120.012758 PMC7573258

[147] Legouis, D., Faivre, A., Cippà, P. E. & de Seigneux, S. Renal gluconeogenesis: an underestimated role of the kidney in systemic glucose metabolism. *Nephrol. Dial. Transplant.* **37**, 1417–1425 (2022). 33247734 10.1093/ndt/gfaa302

[148] Verissimo, T., Faivre, A., Rinaldi, A., Lindenmeyer, M. & de Seigneux, S. Decreased renal gluconeogenesis is a hallmark of chronic kidney disease. *J. Am. Soc. Nephrol.* **33**, 810–827 (2022). 35273087 10.1681/ASN.2021050680 PMC8970457

